# Zinc Oxide‐Graphitic Carbon Nitride Composites: Synthesis, Properties, and Application Scopes in Environmental Remediations

**DOI:** 10.1002/smll.202409637

**Published:** 2025-07-25

**Authors:** Humaira Asghar, Muhammad Saeed, Fatemeh S. Mirsafi, Mustafa K. Ismael, Till Leißner, Yogendra Kumar Mishra

**Affiliations:** ^1^ Smart Materials NanoSYD Mads Clausen Institute University of Southern Denmark Alsion 2 Sønderborg DK‐6400 Denmark; ^2^ Department of Chemistry Government College University Faisalabad Faisalabad 38000 Pakistan; ^3^ Middle Technical University‐Institute of Technology St. 54‐AlZafaranyiah Baghdad 10066 Iraq

**Keywords:** environmental remediation, graphitic carbon nitride, metal oxides, photocatalysis, ZnO‐g‐C_3_N_4_ composites

## Abstract

Environmental pollution constitutes a significant threat to ecosystems and human health, necessitating advanced solutions for its mitigation. This review emphasizes the development and application of ZZnO‐g‐C₃N₄ composites as emerging photocatalysts for environmental remediation. Conventional wastewater treatment methods, although widely used, suffer limitations like low efficacy, high costs, and secondary waste generation, etc. This has led to a growing interest in heterogeneous photocatalysis, offering high efficiency, eco‐friendliness, and affordability. Thus, there is an urgent need to develop innovative nanocomposites with distinctive features such as higher absorption of visible light, efficient charge separation, and excellent stability for the decontamination of pollutants. The coupling of ZnO with g‐C_3_N_4_ composite could enhance visible‐light absorption and promote charge separation, increasing photocatalytic performance. This contribution focuses on environmental challenges and the significance of photocatalysis in the elimination of environmental pollutants. It includes discussions on properties, methods for improving photoactivity, synthesis techniques, and applications of ZnO, g‐C_3_N_4_, and ZnO‐g‐C_3_N_4_ composite materials. Also, this review covers recent advancements in improving the efficiency of these advanced nanocomposites in breaking down organic pollutants in wastewater. It is anticipated that this article will inspire the conceptualization and advancement of different ZnO‐g‐C_3_N_4_ composites for broader applications in materials design, photocatalysis, and water remediation.

## Graphical abstract

Environmental pollution constitutes a significant threat to ecosystems and human health, necessitating advanced solutions for its mitigation. This review focuses on the development and application of ZnO‐g‐C₃N₄ composites as emerging photocatalysts for environmental remediation. The coupling of ZnO with g‐C_3_N_4_ composite can enhance visible‐light absorption and promote charge separation, increasing photocatalytic performance, making this review very interesting for the environmental community.

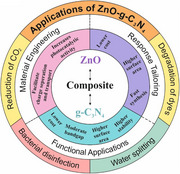

## 1 Introduction

**Figure 1:**
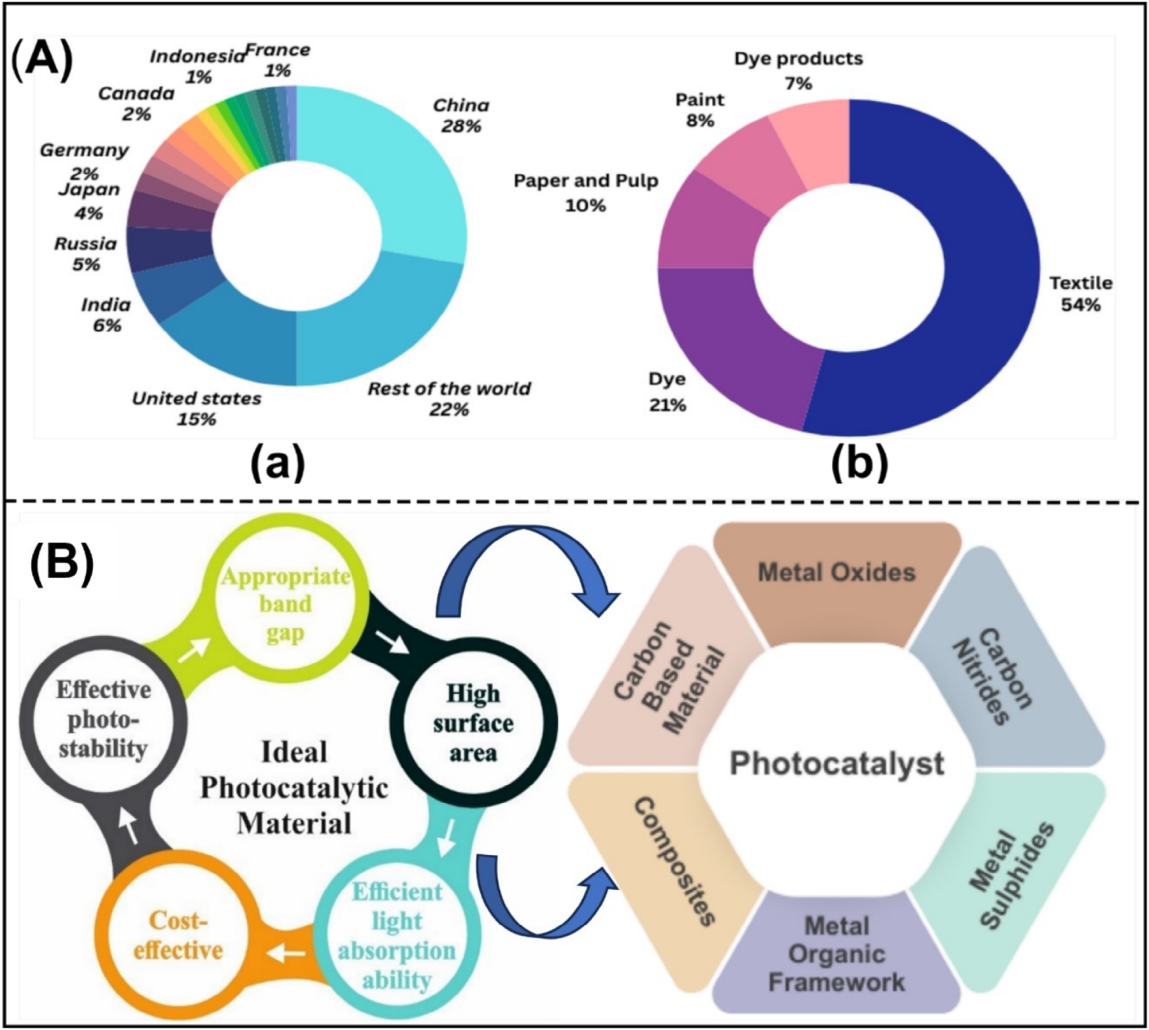
A) Percentage distribution of greenhouse gas emissions (a), Dye pollution by various industries worldwide (b),^[^ ^2^, ^5^ ^]^ B) Pre‐conditions for any material (left) to be an ideal photocatalyst and potential photocatalytic material candidates (right).

Recently, environmental pollution has caused significant harm to human health and the environment. Especially, the combustion of fossil fuels produces large amounts of carbon dioxide, causing global warming. This excessive emission of carbon dioxide has a significant effect on the carbon cycle, causing environmental issues.^[^ ^1^ ^]^ Thus, **Figure** **1**A(a) displays the percentage distribution of greenhouse gas emissions worldwide.^[^ ^2^ ^]^ Furthermore, it causes severe environmental impacts, including frequent and intense weather events such as hurricanes, heat waves, and heavy rainfall. This also leads to the melting of polar ice caps and glaciers, resulting in rising sea levels that threaten the coastal areas and the overall ecosystems. In addition, global warming has disrupted natural habitats and threatened biodiversity to a large extent, causing ecosystem shifts and endangering species. Moreover, the organic contaminants, such as dyes, phenols, and pesticides, found in industrial manure harm human health and the environment.^[^ ^3^ ^]^ Remarkably, some of these are also known as persistent organic contaminants that are even difficult to decontaminate and remain in the environment for a very long period. For instance, the discharge of dyes into wastewater is rapidly increasing due to the higher demand for textiles and consumer goods, making it a serious environmental issue.^[^ ^4^ ^]^ The textile industry is the foremost source of dye effluents, accounting for 54% of the total dye effluents found in the environment worldwide, as shown in Figure 1A (b). This extensive contribution arises from the various methods involved in textile production, such as washing, bleaching, and dying fabrics. Following the textile industries, the dye industries release over 21% of dye effluents. This industry emphasizes dyeing processes applied to textiles and other materials, leading to substantial effluent discharge. The paper and pulp industries are also responsible for releasing ≈10% of dye effluents. The use of dyes in the paper industry for coloring and printing purposes results in significant dye waste. The paint industry contributes 8% of the dye effluents as well. Notably, the paint industry produces dye waste through the application and production of coatings and paint. Finally, the dye manufacturing industry releases ≈7% of the total dye effluents, which includes the waste produced during the manufacture of several types of dyes used across various industries.^[^ ^5^ ^]^ Consequently, the release of these contaminants into water bodies and soil can cause severe ecological damage, disturbing aquatic life, soil health, and possibly human health by contaminating water sources. Thus, it is crucial to develop green and effective techniques to degrade environmental contaminants.^[^ ^6^ ^]^

Remarkably, physical, chemical, and biological approaches have been utilized to decontaminate organic contaminants from the environment. Physical methods include adsorption, membrane filtration, ion exchange, photo‐Fenton process, and nanofiltration. These are ineffective methods for wastewater remediation, as they do not decompose organic contaminants but transfer them from one phase to another.^[^ ^7^ ^]^ In contrast, the biological methods, such as phytoremediation, microbial remediation, mycoremediation, phycoremediation, and bacterial remediation, are slow, and some pollutants are toxic and non‐biodegradable to microorganisms. Chemical methods include chemical reduction, ozonation, photocatalysis, advanced oxidation process, and coagulation/flocculation. These methods have been suggested to cope with environmental pollutants up to some extent.

Among these strategies, the photocatalysis process is regarded as a highly active and green technique for removing organic pollutants from the environment.^[^ ^8^ ^]^ They can effectively use solar energy, and the involved photocatalyst gets excited by absorbing light, generating holes and electrons that trigger the redox reactions.^[^ ^9^ ^]^ Notably, photocatalytic degradation with semiconductor metal oxides is a promising protocol for environmental remediation because it has shown a higher efficiency in removing toxic pollutants from the environment without involving very complex technologies.^[^ ^10^ ^]^ The semiconductor‐based photocatalysis is considered to be a green technique that is used for different environmental applications, such as the degradation of organic contaminants, carbon dioxide reduction, bacterial disinfection, and water splitting.^[^ ^6^, ^11^ ^]^ Moreover, it is considered a promising environmental remediation technology due to the use of abundant solar energy and molecular oxygen as the oxidants. It also decontaminates the pollutants into harmless substances such as water and carbon dioxide.^[^ ^12^ ^]^ Therefore, the search for visible active materials is currently the most important matter. Notably, several semiconductors like g‐C_3_N_4_, TiO_2_, SnO_2_, BiVO_4_, Fe_2_O_3_, SmFeO_3_, CuO, ZnO, and Bi_2_O_3_ have been utilized as photocatalysts in vast applications.^[^ ^13^ ^]^ However, to be an efficient catalyst, the involved material must fulfil certain components as mandatory criteria. The most appealing features for the selection of these components are (a) the superior redox ability of wide bandgap semiconductors and (b) the high photo‐response of narrow‐bandgap semiconductors under visible light.^[^ ^14^ ^]^ The pre‐conditions (left) for any material to be an ideal photocatalyst and potential photocatalytic material candidates (right) are shown by schematics in Figure 1B.

Recently, several photocatalytic nanomaterials, such as ZnO, SnO_2_, TiO_2,_ and CdS, etc. demonstrated higher photocatalytic activities for the treatment of environmental pollutants, but their photocatalytic activities are mostly limited to the UV region, which comprises only 4% of the solar spectrum. The visible light region, which comprises of 46% solar spectrum, is nearly ineffective for these photocatalysts due to their large band gap, limited number of reactive sites on their surface, and higher recombination rates of photoelectron and hole pairs.^[^ ^15^ ^]^ Among several photocatalysts, ZnO has been considered a promising photocatalyst due to its distinctive features, such as lower cost, less toxicity, higher stability, and unique electronic properties. However, its photocatalytic activity is limited due to its large band gap, response to the dominantly UV region, and higher recombination of photoelectrons and hole pairs. Therefore, to overcome these challenges, several strategies have been adopted to increase the photocatalytic activity of pure ZnO nano‐and microscopic materials. These strategies include doping of metals/ nonmetals, noble metals, coupling with transition metal dichalcogenides, and also coupling with narrow band gap semiconductors. Among these approaches, coupling with narrow‐band semiconductor to form a heterojunction has been considered to be an effective strategy for the treatment of environmental pollutants.^[^ ^16^ ^]^ Moreover, the fabrication of heterojunction composites is necessary to absorb sunlight irradiation for photocatalytic applications in environmental remediation.^[^ ^17^ ^]^ Additionally, it can enhance the separation of photogenerated charge carriers and improve the photocatalytic performance of the catalyst.^[^ ^18^ ^]^ For instance, Kole et al.,^[^ ^19^ ^]^ synthesized wurtzite ZnS nanosheets, porous ZnO nanostructures, and ZnS/ZnO nanocomposites utilizing the thermal method. It was exhibited that the ZnS/ZnO composite showed the highest photocatalytic efficiency (74%) for the degradation of methylene blue dye as compared to pristine wurtzite ZnS nanosheets and porous ZnO nanostructures, which showed 53% and 72% degradation efficiency under visible light irradiation. The higher photocatalytic activity under visible light irradiation and visible photoluminescence (PL) emission due to greater surface area and porous nature make these materials highly efficient for future applications in optoelectronic devices.^[^ ^19^ ^]^ The carbon nanostructures decorated ZnO have shown remarkable potential in the direction of photocatalytic dye degradation.^[^ ^20^ ^]^ Similarly, the ZnO‐CND (carbon nanodots) nanocomposite was synthesized through the microwave method, which demonstrated the highest photocatalytic efficiency (≈70%) for the degradation of methylene blue dye in contrast to pure ZnO under visible light irradiation.^[^ ^21^ ^]^ Moreover, the ZnO‐carbon nanofiber has exhibited higher photocatalytic acrolein degradation activity as compared to pristine ZnO and carbon fiber.^[^ ^22^ ^]^

**Figure 2:**
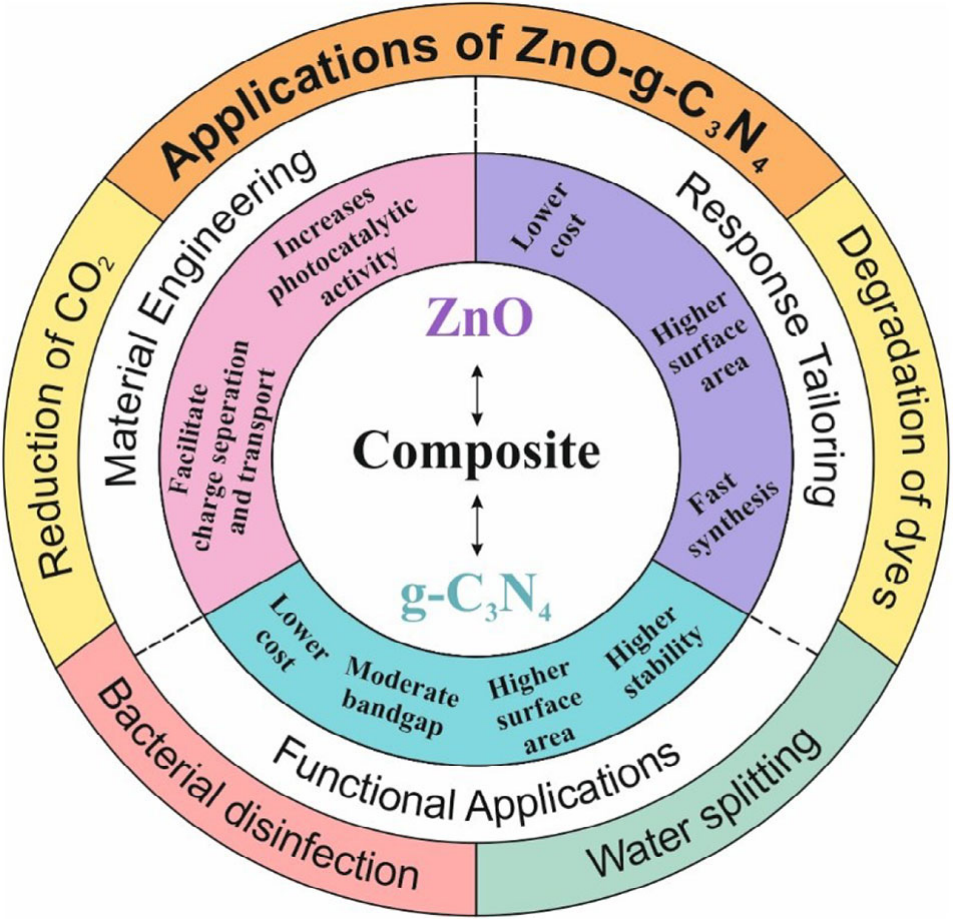
Overview of the ZnO‐g‐C_3_N_4_ composite for environmental applications.

Remarkably, the ZnO‐g‐C_3_N_4_ nanocomposite also delivered an excellent photocatalytic performance, making it a highly promising material for environmental remediation. The graphitic carbon nitride (g‐C_3_N_4_) forms a heterojunction composite with ZnO to lower the recombination rate of photoinduced electrons and holes, which effectively increases the photocatalytic activity of ZnO owing to the suitable conduction and valence bands.^[^ ^23^ ^]^ Due to these advantages, the ZnO/g‐C_3_N_4_ based heterostructures have been synthesized by the community for the treatment of environmental pollutants. For instance, Shemeena and Binitha synthesized a ZnO‐g‐C_3_N_4_ nanocomposite by a sol–gel approach, which showed a maximum photocatalytic activity for decontaminating Congo red in contrast to pure ZnO and g‐C_3_N_4_.^[^ ^24^ ^]^ The heterojunction formation, higher surface area accessibility, and an appropriate bandgap, etc., actually boosted the photocatalytic activity of the ZnO‐g‐C_3_N_4_ composite.^[^ ^14b^ ^]^ Despite of huge potential of ZnO‐g‐C_3_N_4_ composite in environmental remediation, there exist very few reports in the literature, which motivated us to formulate this contribution to trigger the photocatalytic potential of ZnO‐g‐C_3_N_4_ material in the community. This review primarily focuses on the recent advancements and innovative strategies for enhancing the photocatalytic performance of ZnO/g‐C_3_N_4_ composites for environmental remediation (**Figure** **2**); however, its role in bacterial disinfection and water splitting is also covered.

## 2 ZnO as an Emerging Photocatalytic Material

**Figure 3:**
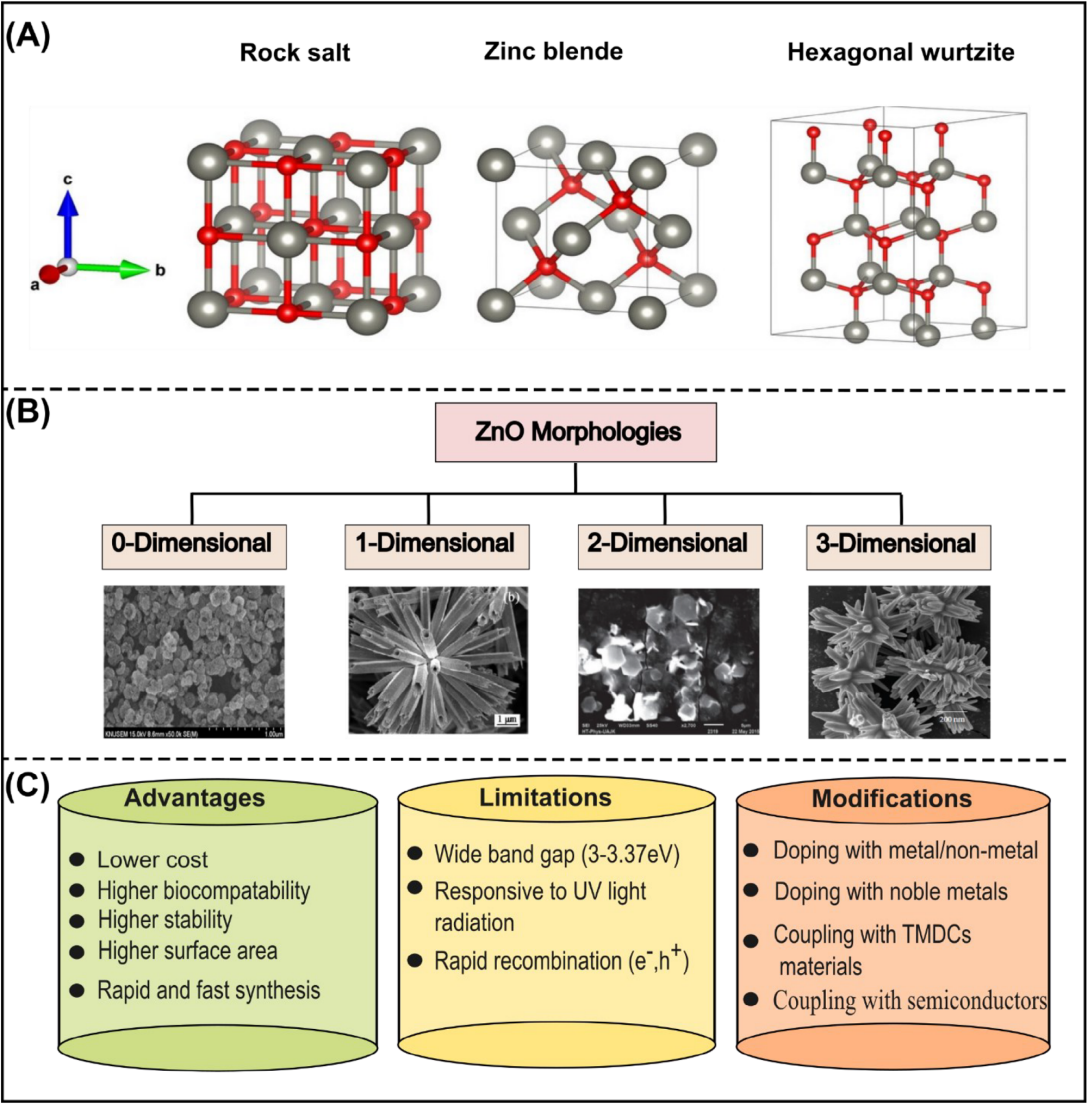
A) Crystal structures of ZnO. Reproduced under the terms of the CC BY 4.0 license from^[^ ^31^ ^]^ Copyright 2023, Wiley‐VCH. B) Different morphologies of synthesized ZnO based on dimensions: Reproduced with permission from^[^ ^27a^ ^]^ Copyright 2015, Elsevier. Reproduced with permission from^[^ ^27d^ ^]^ Copyright 2007, American Chemical Society. Reproduced with permission from^[^ ^27c^ ^]^ Copyright 2018, Elsevier. Reproduced under the terms of the CC BY 4.0 license from^[^ ^27b^ ^]^ Copyright 2016, Wiley‐VCH.^[^ ^27b^ ^]^ (C) Advantages, limitations, and modifications of ZnO.

Notably, several semiconductors such as TiO_2_, SnO_2_, BiVO_4_, Fe_2_O_3_, SmFeO_3_, CuO, ZnO, and Bi_2_O_3_ have been utilized as photocatalysts in many applications.^[^ ^25^ ^]^ In recent years, ZnO has gained significant attention as an emerging photocatalyst due to its unique structural characteristics and exceptional properties, particularly for environmental remediation. ZnO typically adopts a cubic crystal structure (zinc blende), rock salt, or hexagonal wurtzite arrangement. The stability of the zinc‐blende form of ZnO requires specific conditions, whereas the rock salt structure is rare and forms under high pressure. The wurtzite form, however, exhibits the highest thermodynamic stability among these structures.^[^ ^26^ ^]^ The different crystal structures of ZnO are shown in **Figure** **3**A. Moreover, based on nanoscale dimension, ZnO adopts several morphologies like 0D (e.g., nanoparticles), 1D (e.g., nanorods, nanowires, nanotubes), 2D (e.g., nano‐disks, nanoplates, nanosheets), and 3D (e.g., nanoflowers, tetrapods, multipods, nanoforests). Almost any structural geometry of nanostructure one can imagine is possible with ZnO material; it all depends upon the involved synthesis method, parameter control, reproducibility, and upscalability. Just some exemplary ZnO micro‐ and nanostructure morphologies are shown in Figure 3B.^[^ ^27^ ^]^ Remarkably, ZnO has extensive applications in various industries, such as chemicals, electronics, space engineering, medicine, gas sensors, transducers, and electrodes. It is extensively utilized as a stabilizer in several materials, including glass, plastics, cement, lubricants, ceramics, paints, ointments, sealants, batteries, colors, adhesives, foods, ferrites, and fire retardants.^[^ ^28^ ^]^ ZnO is regarded as an excellent photocatalyst due to its distinctive characteristics, like higher stability, higher biocompatibility, higher surface area, rapid synthesis, and cost‐effectiveness.^[^ ^29^ ^]^ However, some of its properties, like a wide bandgap (3–3.37 eV), response to UV light radiation, and rapid recombination of electrons and holes, hinder its desired photocatalytic activity in the visible region.^[^ ^30^ ^]^ The higher bandgap of ZnO is also a kind of barrier for its synthesis under visible light irradiation. Figure 3C displays the advantages, limitations, and modification techniques used to enhance the photocatalytic activity of ZnO material.

### 2.1 Methods to Increase the Photocatalytic Activity of ZnO

The modification of ZnO to overcome these limitations is one of the most extensively researched topics in photocatalysis. Several methods have been proposed for increasing the activity of ZnO nano‐microstructures. These include doping with metals or non‐metals, coupling with semiconductors, and noble metal deposition.^[^ ^32^ ^]^

#### 2.1.1 Doping with Metals or Non‐metals

The foremost challenge hindering the practical use of ZnO is its tendency for rapid recombination of photoinduced charge carriers and its partial sensitivity to visible light. Typically, the ZnO exhibits a wide gap (≈3.37 eV) that restricts its light absorption to UV wavelengths mainly, which represent only a small fraction of solar radiation. This UV‐exclusive absorption significantly constrains the utilization of solar energy to activate photocatalytic reactions. To address this limitation, doping with metal or nonmetal ions is employed to enhance the photocatalytic ability of ZnO to absorb visible light, as discussed in recent reviews. Incorporating metal or nonmetal atoms through doping can alter the photoelectric characteristics of ZnO, enabling it to absorb visible light more effectively by reducing its bandgap.^[^ ^33^ ^]^ Furthermore, the doping induces the creation of vacancies like interstitial or substitutional defects, leading to modifications in the conduction, color, and magnetic and optical characteristics of doped oxides.^[^ ^34^ ^]^ In a previous work, the Ag‐doped ZnO was synthesized using a reflux chemical approach and investigated for the degradation of methylene blue and rose Bengal dyes, respectively. The 5‐Ag‐ZnO showed degradation efficiency of 96% and 98% against rose Bengal and methylene blue dyes in 30 min, respectively. Especially, the reduction in the band gap of ZnO is due to the incorporation of Ag into ZnO nanoparticles, which thereby increases the photocatalytic activity as compared to pristine ZnO. Moreover, the antifungal study was also carried out, which showed 45% efficiency for the 7‐Ag‐ZnO system.^[^ ^35^ ^]^ Similarly, Xie et al.,^[^ ^36^ ^]^ reported the synthesis of Phosphorus‐doped ZnO nanocombs via the chemical vapor deposition (CVD) approach, which were utilized for the degradation of methylene blue dye, and they demonstrated an improved photocatalytic activity against methylene blue degradation. Recently, several literature works have discussed the enhancement of the photocatalytic activity of ZnO by doping with metal and nonmetal ions.^[^ ^37^ ^]^

#### 2.1.2 Doping with Noble Metals

Doping ZnO with noble metal is a substantial factor for enhancing its photocatalytic performance. Notably, the noble metals are widely known as storage for photoinduced electrons and holes.^[^ ^38^ ^]^ It has been illustrated that the noble metals can effectively absorb visible light radiation because of the localized surface plasmon resonance (LSPR) phenomenon.^[^ ^39^ ^]^ This phenomenon arises from the synchronized oscillations of surface electrons, presenting an excellent avenue for increasing the light absorption spectrum of wide‐bandgap semiconductors to the visible range and enhancing their photocatalytic activity.^[^ ^40^ ^]^ Recently, a noble metal‐modified ZnO hybrid system has been reported for the efficient degradation of organic pollutants. The improved characteristics of noble metal–ZnO nanocomposites suggest their potential application as sensors, converters, energy generators, and photocatalysts instead of conventional ZnO materials.^[^ ^41^ ^]^ Various micro‐ and nanoscale structures of noble metal–ZnO nanocomposites have been reported, highlighting their versatility in nanotechnology and related applications. For instance, Guy et al.,^[^ ^42^ ^]^ demonstrated that doping ZnO with noble metals markedly enhances its photocatalytic performance. Their findings indicated that the integration of noble metals into ZnO lowers the recombination rate of photoinduced electrons and holes upon light absorption, thereby improving the photocatalytic efficiency of ZnO micro and nanostructures.

#### 2.1.3 Coupling with Transition Metal Dichalcogenide Materials

The photocatalytic activity of pure ZnO can be enhanced by coupling with 2D transition metal dichalcogenides (TMDCs) materials. Recently, the transition metal dichalcogenides (MoS_2_, WS_2,_ etc.) have attained significant attention due to their unique properties, such as higher surface area, narrow band gap, layered structure, cost‐effectiveness, and greater mechanical strength, which play an important role in increasing the photocatalytic activity.^[^ ^43^ ^]^ Mohammed et al.,^[^ ^44^ ^]^ reported the synthesis of ZnO/MoS_2_ nanocomposites by the hydrothermal approach and investigated the effect of TMDCs incorporation into ZnO for the degradation of methylene blue, malachite green, and crystal violet dyes, respectively. Moreover, the ZnO/MoS_2_ was also investigated for the degradation of sulfamethoxazole, meloxicam, and trimethoprim antibiotics. It was demonstrated that the prepared ZnO/MoS_2_ nanocomposite exhibited higher photocatalytic degradation efficiency (≈100%) against malachite green, crystal violet, methylene blue, sulfamethoxazole, trimethoprim, and meloxicam as compared to pristine ZnO nanostructures. Similarly, Cantarella et al.,^[^ ^45^ ^]^ prepared ZnO/MoS_2_/PMMA nanocomposites via a simple solution and sonication method. It was reported that the ZnO/MoS_2_/PMMA nanocomposites displayed the highest photocatalytic activity for the degradation of SDS and RhB in contrast to pure ZnO nanostructures. Several research works have already demonstrated the importance of transition metal dichalcogenides in environmental remediation.^[^ ^46^ ^]^

#### 2.1.4 Coupling with Semiconductor Materials

The construction of heterojunctions by coupling two or more semiconductors with suitable band gaps is an effective method for developing visible‐light active photocatalysts. Heterojunctions are promising photocatalysts with higher photocatalytic efficiency because of visible light absorption and the rate of recombination of excitons.^[^ ^14^, ^17^, ^47^ ^]^ Heterojunctions are promising photocatalysts with greater photocatalytic efficiency due to their lower rate of recombination of excitons and higher absorption of visible light.^[^ ^48^ ^]^ Notably, coupling ZnO with other semiconductors with smaller band gaps to create a heterojunction can significantly enhance its photocatalytic performance. This strategy not only improves the separation of photoinduced electron‐hole pairs, but also extends light absorption to the visible range.^[^ ^14^, ^32^, ^49^ ^]^ Consequently, the development of heterojunction nanocomposites has emerged as a promising and viable method to address the limitations of pure ZnO in terms of photocatalytic performance.^[^ ^14^, ^50^ ^]^ However, certain challenges hinder the practical application of heterojunctions as photocatalysts, and they are: i) the synthesis of non‐toxic, low‐cost heterojunctions with high photo response and redox ability, and ii) the separation and recycling of heterojunctions.^[^ ^47^ ^]^ Interestingly, the coupling of ZnO with g‐C_3_N_4_ has been found to be an efficient approach to enhance the photocatalytic activities of zinc oxide nanomaterials. The conjugated framework of g‐C_3_N_4_ significantly reduced the recombination rate of the photoinduced holes and electrons, which further facilitated the charge separation and transport. Conversely, the formation of an electron‐donor acceptor complex at the zinc oxide and graphitic carbon nitride interface leads to the creation of a heterojunction with a lower charge carrier recombination rate and increased photocatalytic performance in contrast to pure ZnO material.

## 3 Graphitic Carbon Nitride (g‐C_3_N_4_)

**Figure 4:**
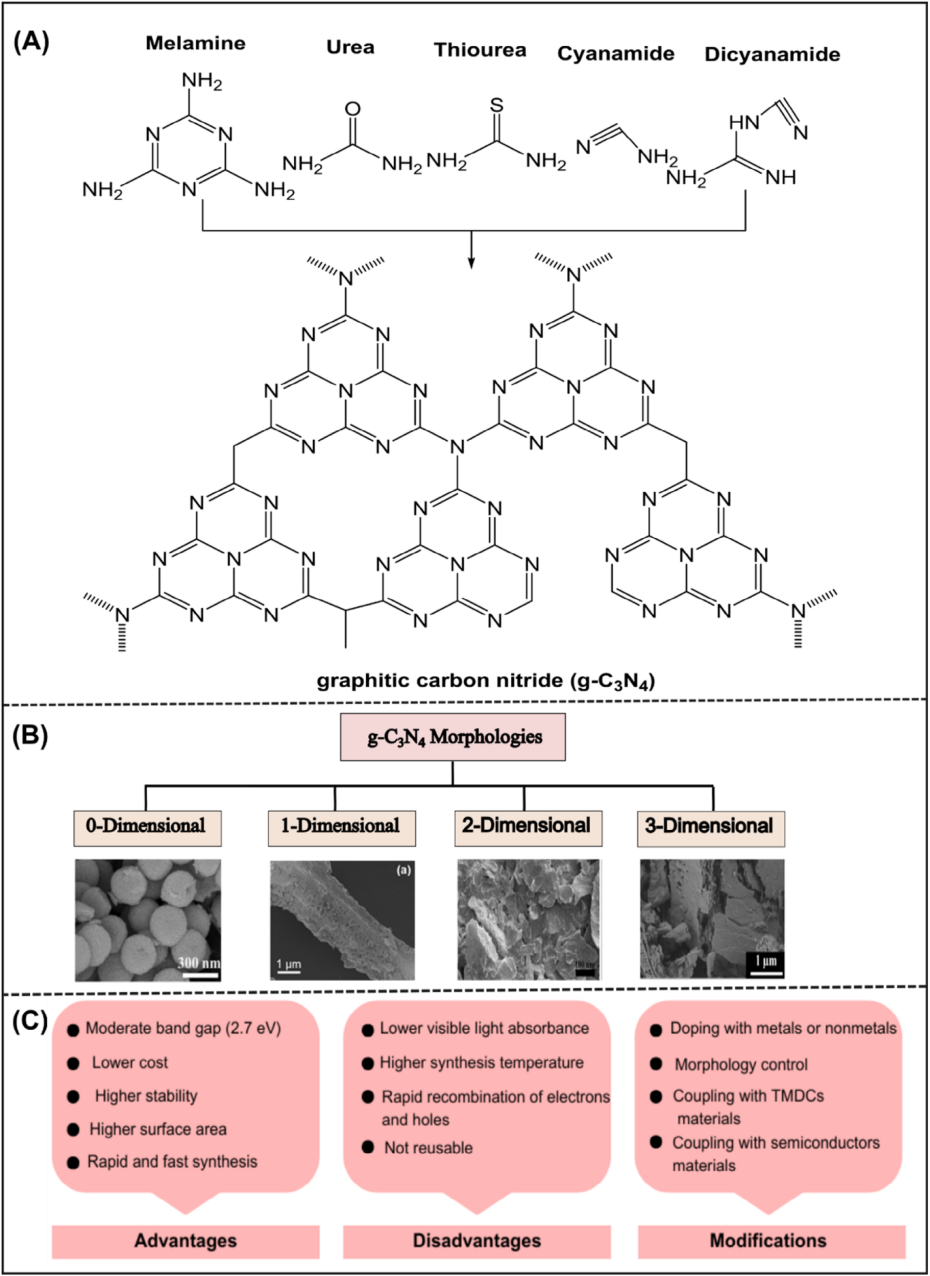
A) Structure and synthesis of g‐C_3_N_4_. B) Several synthesized morphologies of g‐C_3_N_4_ based on dimensions: Reproduced under the terms of the CC BY 4.0 license from^[^ ^57^ ^]^ Copyright 2023, MDPI. Reproduced with permission from^[^ ^58^ ^]^ Copyright 2024, Elsevier. Reproduced with permission from^[^ ^59^ ^]^ Copyright 2017, Springer Nature. Reproduced with permission from^[^ ^60^ ^]^ Copyright 2020, John Wiley and Sons. C) Advantages, disadvantages, and modifications of g‐C_3_N_4_.

A narrow bandgap semiconductor can be utilized as a photocatalyst in visible light irradiation, although the rapid recombination of photo‐induced holes and electrons hinders its photocatalytic activity. Several efforts have been made to prevent the recombination of holes and electrons by creating nanocomposite materials.^[^ ^49^, ^51^ ^]^ Various research works have reported the preparation of visible‐active photocatalysts for wastewater treatment. Notably, the development of visible‐active materials for the treatment of environmental contaminants is considered an important issue among researchers with respect to photocatalysis.^[^ ^18^ ^]^ In the broad family of graphitic carbon nitride based semiconductors, the polymeric graphitic carbon nitride (g‐C_3_N_4_), the most stable allotrope of carbon nitride, is considered an appropriate material for visible‐active photocatalysts owing to its following exceptional features: facile synthesis, low cost, moderate energy gap (around 2.7 eV), high stability, and high surface area ^[^ ^17^, ^47^, ^49^, ^52^ ^]^ Moreover, different methods have been employed to synthesize g‐C_3_N_4_, including thermal condensation, solvothermal synthesis, CVD, and electrochemical methods. Generally, the g‐C_3_N_4_ is produced by thermal condensation using inexpensive nitrogen materials such as urea, dicyandiamide, melamine, and cyanamide, and this method is very promising because of its cost‐effective precursors.^[^ ^53^ ^]^ Remarkably, Urea is favored for producing a thin layer of g‐C_3_N_4_ with a large surface area, making it a popular choice for many synthesis methods. The synthesis temperature depends on the specific precursor used, and the duration of pyrolysis determines the thickness of the resulting g‐C_3_N_4_ layers. This adaptability in synthesis methods and precursor selection greatly contributes to optimizing g‐C_3_N_4_ properties for diverse applications, including photocatalysis and pollutant degradation.^[^ ^54^ ^]^ Wang et al., first developed graphitic carbon nitride for hydrogen production, and they found it to be a useful material for extensive photocatalytic applications.^[^ ^55^ ^]^ It has attracted enormous attention over the last decades and has emerged as an ideal candidate for CO_2_ reduction, H_2_O splitting, reduction, and pollutant decontamination. As an innovative semiconductor, the graphitic carbon nitride is relatively different from other photocatalysts as it can simply be altered to form composite materials with certain compositions, higher surface area, refined morphologies, and porous nature.^[^ ^56^ ^]^ The molecular structures of the different precursors used for the synthesis of graphitic carbon nitride (g‐C_3_N_4_) and possible structural variants are shown in **Figure** **4**A,B, respectively.

### 3.1 Methods to Increase the Photocatalytic Activity of g‐C_3_N_4_

The g‐C_3_N_4_ implications in the field of photocatalysis have been recognized in a substantial number of research papers published in recent years, covering a wide range of applications. Despite many novel advantages, it still has limitations that strongly influence its photocatalytic activity. The most common limitations detected during photocatalysis with common g‐C_3_N_4_ materials include the higher recombination rate of photogenerated electrons and hole pairs, lower specific surface area, partial visible‐light absorption, and lower absorption coefficient.^[^ ^61^ ^]^ Several methods have been used to improve the photocatalytic activity of g‐C_3_N_4_. These methods include doping with metals or nonmetals, controlled morphology, and coupling with other semiconductors. Considering the significance of graphitic carbon nitride, the advantages, disadvantages, and potential modifications of g‐C_3_N_4_ are described in Figure 4C.

### 3.2 Doping with Metals or Non‐Metals

Doping involves the addition of foreign impurities into the framework of g‐C_3_N_4_ to improve its optical, physical, and electrical properties. In the field of photocatalysis, doping plays a significant role in moderating the absorption of light and the redox potential for numerous photocatalytic applications. The doping of metals imparts several advantageous properties to g‐C_3_N_4_, such as reducing the bandgap and increasing light absorption, thereby improving its photocatalytic activity. Notably, to introduce metal ions into g‐C_3_N_4_, a soluble metal salt was mixed with the g‐C_3_N_4_ precursor and dissolved in distilled water with stirring to confirm a uniform distribution. Li and co‐workers in their research confirmed that the Er doping narrows down the bandgap and increases the photocatalytic activity of g‐C_3_N_4_.^[^ ^62^ ^]^ Moreover, the doping with several nonmetals like O, P, S, F, Cl, Br, and I was found to be a significant approach to increase the activity of g‐C_3_N_4_. Hung et al., reported that P‐ and O‐doped g‐C_3_N_4_ exhibited a 6.2 times higher degradation rate of enrofloxacin than pristine g‐C_3_N_4_.^[^ ^63^ ^]^

### 3.3 Morphology Control

The g‐C_3_N_4_ exhibits several morphologies owing to its flexible structure. The controlled morphologies play an important role in enhancing the photocatalytic activity of g‐C_3_N_4_ by facilitating efficient charge transportation and migration during the photocatalytic reaction. The different morphologies of g‐C_3_N_4_ have been synthesized, like 0D (hollow spheres), 1D (nanowires, nanotubes, nanorods), 2D (nanosheets), and 3D (nanospheres, hydrogels, and aerogels). Figure 4B shows the morphologies of several synthesized g‐C_3_N_4_.^[^ ^57^, ^58^, ^59^, ^60^ ^]^ For instance, Liu et al., demonstrated in their research that g‐C_3_N_4_ nanorods exhibit higher photocatalytic activity for the decontamination of RhB and water splitting.^[^ ^64^ ^]^ Similarly, Nabi et al., demonstrated that g‐C_3_N_4_ nanorods exhibit improved photocatalytic activity for the photodegradation of MO, RhB, and MB dyes.^[^ ^65^ ^]^ Moreover, Shi et al. prepared spherical mesoporous g‐C_3_N_4_, and the results showed that the spherical mesoporous g‐C_3_N_4_ displayed greater photocatalytic activity for the degradation of RhB under visible light irradiation than bulk g‐C_3_N_4_.^[^ ^66^ ^]^

### 3.4 Coupling with TMDCs

The incorporation of TMDCs into g‐C_3_N_4_ plays a significant role in enhancing the photocatalytic activity of g‐C_3_N_4_. Remarkably, the 2D TMDCs comprise transition metals sandwiched between the two layers of chalcogens, which are coupled with graphitic carbon nitride to increase their photocatalytic activity.^[^ ^67^ ^]^ Senthilnathan et al.,^[^ ^68^ ^]^ reported the synthesis of MoS_2_‐g‐C_3_N_4_ nanocomposites using the hydrothermal method and exhibited a higher photocatalytic activity for the degradation of methylene blue under sunlight as compared to pure g‐C_3_N_4_. The main factor in the enhancement of its photocatalytic activity is the incorporation of MoS_2_ into g‐C_3_N_4,_ which results in a higher absorption of visible light to generate a large number of electrons and holes. This leads to an increase in the number of active sites, which can absorb more methylene blue.^[^ ^68^ ^]^ Moreover, Xin et al.,^[^ ^69^ ^]^ studied the synthesis of g‐C_3_N_4_/MoS_2_ heterojunction through a ball milling approach. The results indicated that the g‐C_3_N_4_/MoS_2_‐2% heterojunction displayed ≈4.3 times higher photocatalytic activity for the degradation of RhB dye as contrast to bulk g‐C_3_N_4_. It was reported that the increase in the photocatalytic activity was due to effective charge separation and migration of photoelectrons and hole pairs in the heterojunction.

### 3.5 Coupling with Semiconductor Materials

The rapid recombination of photogenerated electrons and holes limits the efficiency of the photocatalytic reaction, thus limiting its practical applications. Therefore, g‐C_3_N_4_ was coupled with another semiconductor material to address this issue to form a heterojunction. This not only improves the separation of photoinduced electrons and holes but also increases the absorption range of visible light.^[^ ^70^ ^]^ Thus, g‐C_3_N_4_‐based materials can successfully enhance their activity and are extensively utilized in wastewater treatment because of their excellent physical, optical, and electrical properties. There are many reviews on g‐C_3_N_4_‐based photocatalysts to which readers can refer.^[^ ^56^, ^71^ ^]^ Herein, this review mainly focuses on ZnO‐g‐C_3_N_4_ for environmental applications.

### 3.6 Role of Heterojunction Systems

Additionally, there are five types of heterojunction photocatalytic systems, i.e., type‐I, type‐II, Z‐scheme, p‐n, and Schottky heterojunction systems. Mostly, the ZnO‐g‐C_3_N_4‐_based heterojunction shows type‐II and Z‐scheme heterojunctions for charge carrier separation. In type‐II heterojunctions, the two semiconductors (A & B) with overlapping band gaps are combined to develop a stable heterojunction. The valence band position of the semiconductor A exceeds that of semiconductor B. Consequently, electrons move from the conduction band of semiconductor A to that of semiconductor B. Therefore, the increased separation of electrons and holes reduces their recombination rate. The construction of type‐II heterojunctions is extremely desirable for a wide range of photocatalytic applications.^[^ ^72^ ^]^ Another type of heterojunction is the Z‐scheme heterojunction, initially suggested by Bard and Fox.^[^ ^73^ ^]^ In this heterojunction, the electrons produced in the conduction band of semiconductor B are transferred to semiconductor A, where they interact with photoinduced holes. This mechanism helps in reducing the recombination rate of charge carriers by increasing the separation of electrons and holes. Most importantly, different migration patterns of holes and electrons are developed by electric fields produced through different semiconductor bandgap positions (conduction and valence band dissimilarities). Notably, based on the electric field, the charge carriers move to minimize the energy of the system. Therefore, the heterojunctions regulate the migration of holes and electrons.^[^ ^72^ ^]^ Consequently, each type of heterojunction controls the movement of charge carriers, making all types suitable for photocatalytic applications.

## 4 ZnO‐g‐C_3_N_4_ Composite as an Advanced Photocatalytic Material

The ZnO‐g‐C_3_N_4_ composites have emerged as a promising material for environmental applications due to their unique characteristics and exceptional performance. The combination of ZnO and g‐C_3_N_4_ results in a synergistic effect that enhances their photocatalytic activity, making them highly efficient for degrading various pollutants and contaminants. In recent years, the utilization of ZnO‐g‐C_3_N_4_ composites has expanded across various environmental remediation applications, including air purification and wastewater treatment. Here, the diverse developments and extensive applications of ZnO‐g‐C_3_N_4_‐based composites are discussed, which have demonstrated significant importance across vast fields due to their unique properties and versatile functionalities.

### 4.1 Synthesis of ZnO‐g‐C_3_N_4_ Composites

Several methods have been reported for the synthesis of ZnO‐g‐C_3_N_4_ composites for the treatment of environmental pollutants. For instance, Grish et al.,^[^ ^74^ ^]^ synthesized ZnO‐g‐C_3_N_4_ by mixing the solution of zinc oxide and graphitic carbon nitride in distilled water. The mixture was stirred for 6 h at 80 °C. ZnO to g‐C_3_N_4_ ratios of 1:1,1:5, and 5:1 and used to investigate the effect of the catalyst amount on the photocatalytic performance. It was concluded that 5:1 ratio of ZnO‐g‐C_3_N_4_ composite exhibited higher efficiency for dye decontamination and hydrogen development than a composite with a 1:5 ratio.^[^ ^74^ ^]^ The hydrothermal method has several advantages, such as low temperature, rapid synthesis, and high purity, and it is also considered an innovative technique for the manufacture of ZnO‐based composites. The ZnO‐g‐C_3_N_4_ was effectively fabricated using a hydrothermal approach for the decontamination of ciprofloxacin under sunlight.^[^ ^75^ ^]^ In addition, Gayathri et al.,^[^ ^76^ ^]^ used a thermal condensation approach to fabricate ZnO/g‐C_3_N_4_ composites using melamine and zinc acetate dihydrate as the precursor. Sodium hydroxide was added to form a precipitate, which was washed and dried to obtain the required sample. Paul et al.,^[^ ^77^ ^]^ prepared the ZnO‐g‐C_3_N_4_ through thermal polymerization of zinc carbonate basic dihydrate and urea for environmental applications. In addition, the coprecipitation approach is considered one of the most appropriate methods for the development of ZnO‐g‐C_3_N_4_ composites due to several advantages, such as simple processing, cost‐effectiveness, and fast reaction time. Garag et al., synthesized the ZnO‐g‐C_3_N_4_ through a co‐precipitation method, which significantly improved its photocatalytic activity for the degradation of Bisphenol A.^[^ ^78^ ^]^ Qin and his co‐workers used the calcination approach for the synthesis of the ZnO‐g‐C_3_N_4_ composite using zinc acetate and urea as precursors. The composite was then investigated for the photodegradation of MO and MB, which exhibited higher photocatalytic performance than pristine ZnO and g‐C_3_N_4_.^[^ ^79^ ^]^

### 4.2 Mechanism Involved in Photocatalysis

Semiconductor‐based heterogeneous photocatalysis at room temperature is highly effective in eliminating contaminants present in wastewater without producing toxic by‐products.^[^ ^80^ ^]^ The degradation mechanism of ZnO‐g‐C_3_N_4_ involves five main steps: Light absorption, charge separation, surface reaction, formation of reactive species, and decomposition of organic pollutants that leverage the unique properties of both materials.

**Figure 5:**
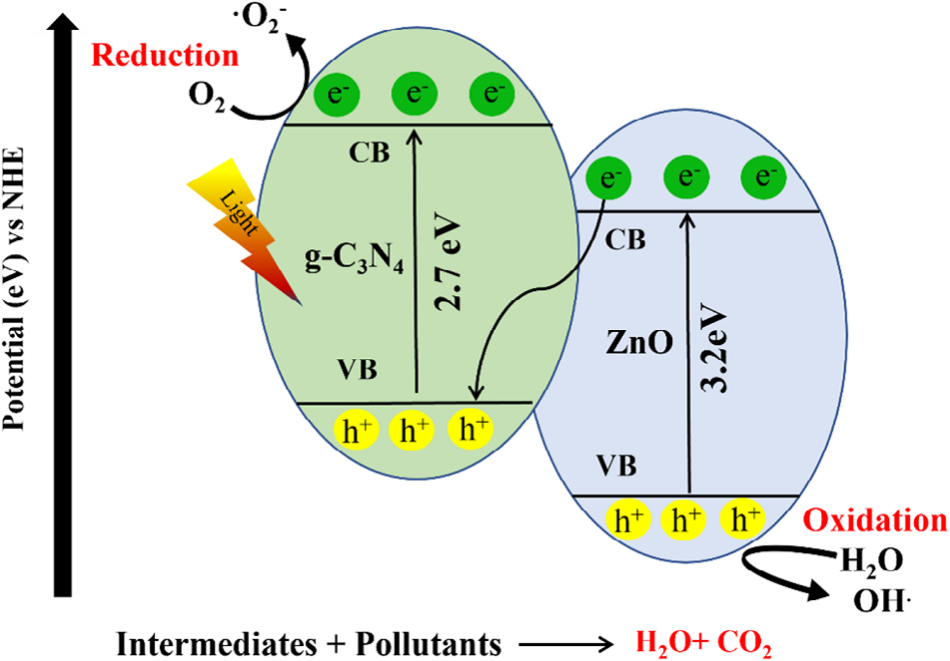
Schematic illustration for the Z‐scheme photocatalytic mechanism of ZnO‐g‐C_3_N_4_.

Initially, upon exposure to light, the ZnO generates electron‐hole pairs due to its inherent semiconducting nature. These electrons and holes migrate to the surface of g‐C_3_N_4_, which acts as a co‐catalyst. Here, the holes in ZnO and electrons in g‐C_3_N_4_ participate in redox reactions with organic pollutants present in the environment. Specifically, the holes in ZnO can easily oxidize organic compounds by breaking them down into smaller and less harmful molecules. Meanwhile, the electrons from g‐C_3_N_4_ can reduce certain pollutants, further aiding their degradation.^[^ ^81^ ^]^ The typical involved Z‐scheme photocatalytic mechanism in ZnO‐g‐C_3_N_4_ heterojunction is illustrated in **Figure** **5**. Additionally, the ZnO‐g‐C_3_N_4_ heterojunction facilitates electron and hole pair separation, preventing their recombination and boosting the overall photocatalytic efficiency. This synergetic interaction between ZnO and g‐C_3_N_4_ causes effective degradation of organic pollutants, highlighting the potential of this heterojunction as a promising candidate for environmental remediation applications.

## 5 Applications

### 5.1 Photocatalytic Degradation of Dyes

**Figure 6:**
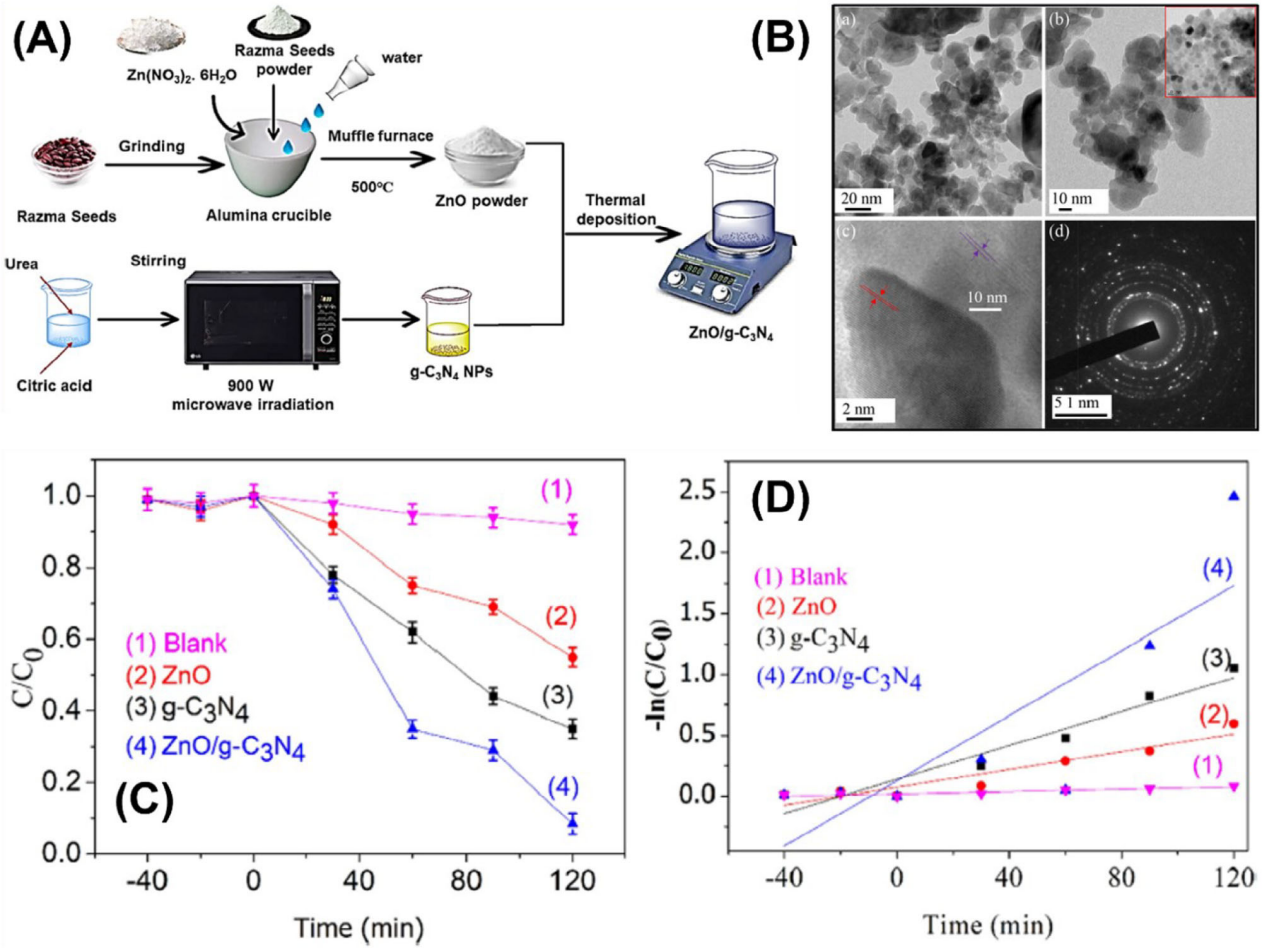
A) Schematic diagram of ZnO/g‐C_3_N_4_ synthesis by the solution‐phase method. B (a,b) TEM image, B (c) HRTEM image, B (d) selected area electron diffraction pattern of ZnO/g‐C_3_N_4_. C) The degradation rate of methylene blue in the sunlight, blank carried out under dark conditions by pristine ZnO, g‐C_3_N_4,_ and ZnO/g‐C_3_N_4_. D) Kinetic simulation studies of ZnO, g‐C_3_N_4,_ and ZnO/g‐C_3_N_4_. Reproduced with permission from^[^ ^74^ ^]^ Copyright 2023, Elsevier.

Dye‐containing effluents from industries can lead to water pollution by altering the chemical composition and biological balance of the aquatic environment. Some dyes resist degradation and can persist in water systems for extended periods, thereby posing risks to aquatic life and human health. Furthermore, the use of certain dyes involves hazardous chemicals and processes, leading to occupational health hazards for workers in dye‐manufacturing facilities. Exposure to dyes and their derivatives has been linked to respiratory problems, skin irritation, and potential long‐term health effects. Landfills and incineration of dye waste may release harmful substances into the air, soil, and groundwater, contributing to environmental degradation and pollution.^[^ ^82^ ^]^ Therefore, it is necessary to synthesize an effective photocatalyst for the photocatalytic decontamination of dyes to protect the environment from pollution. Among photocatalysts, ZnO‐g‐C_3_N_4_ composites have gained significant attention for their superior photocatalytic properties and potential applications in dye degradation. These composites combine the unique properties of zinc oxide and graphitic carbon nitride, offering enhanced catalytic performance, higher stability, and greater selectivity for dye degradation.^[^ ^83^ ^]^ Several synthesis methods have been reported for the development of ZnO‐g‐C_3_N_4_ composite.^[^ ^84^ ^]^ For instance, Grish et al.,^[^ ^74^ ^]^ developed a ZnO‐g‐C_3_N_4_ heterojunction using a solution‐phase synthesis method (**Figure** **6**A). This composite exhibited distinct nanoparticle characteristics along with extended light absorption to the visible range, indicating that it is an effective photocatalyst for degrading methylene blue (MB) dye under simulated sunlight. Specifically, researchers have investigated its efficiency in degrading methylene blue (MB) dye under simulated sunlight conditions. The TEM images of ZnO/g‐C_3_N_4_ [Figure 6B (a,b)] revealed that the composite consisted of aggregated nanoparticles with sizes varying from 50 to 200 nm. Consequently, [Figure 6B (c)] shows the HRTEM image of the composite with two lattice fringes with d‐spacings of 0.26 and 0.335 nm, respectively. The selected area electron diffraction pattern of ZnO/g‐C_3_N_4_ is displayed in [Figure 6B (d)]. Results exhibited that pure ZnO and g‐C_3_N_4_ achieved ≈71% and 52% MB degradation, respectively, within 120 min. In contrast, the ZnO/g‐C_3_N_4_ composite displayed a significantly higher degradation rate, reaching ≈92% MB degradation within the same duration (Figure 6C). Moreover, the composite shows a higher pseudo‐first‐order rate constant (20 × 10^−3^ min^−1^) in contrast to pristine g‐C_3_N_4_ and ZnO (Figure 6D).

**Figure 7:**
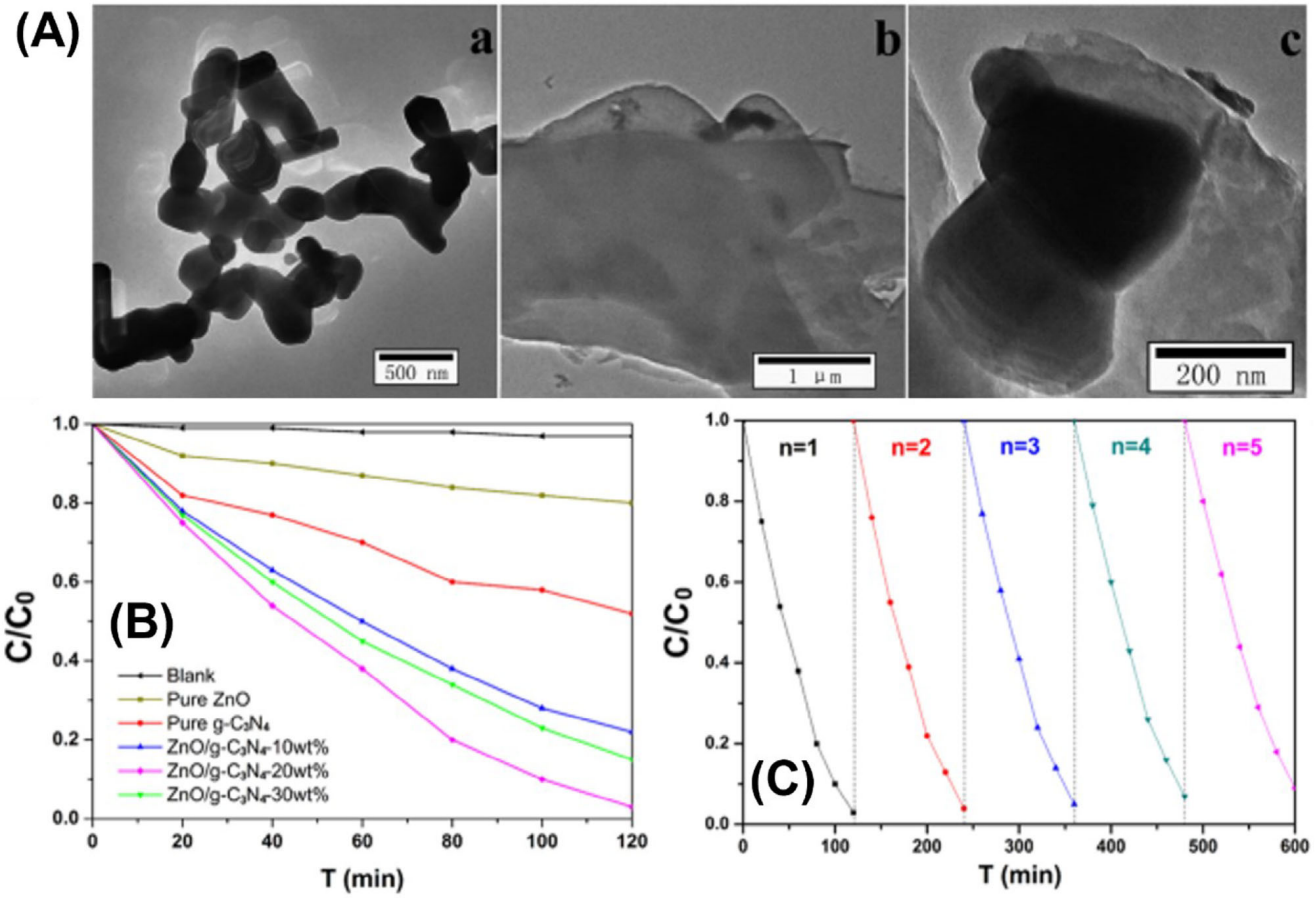
A) TEM images (a) ZnO (b) g‐C_3_N_4_ (c) ZnO/g‐C_3_N_4_. B) Photocatalytic activity of ZnO, g‐C_3_N_4,_ and ZnO/g‐C_3_N_4_ for the decontamination of methyl orange. C) Cycle curve for methyl orange decontamination using optimized ZnO/g‐C_3_N_4_‐20%. Reproduced under the terms of the CC‐BY‐NC 4.0 license from^[^ ^85^ ^]^ Copyright 2019, American Chemical Society.

Similarly, Guan et al.,^[^ ^85^ ^]^ developed highly distributed ZnO/g‐C_3_N_4_ composites with varying amounts of g‐C_3_N_4_. These composites exhibit outstanding photocatalytic activity and durability when used to degrade methyl orange in solution. The researchers investigated the optimal introduction of g‐C_3_N_4_ and the mechanism behind the improved photocatalytic performance, presenting a promising material for organic pollutant removal. Consequently, the TEM images of ZnO and g‐C_3_N_4_ [**Figure** **7**A (a,b)] showed a random arrangement of particles. However, the TEM image [Figure 7A (c)] of ZnO/g‐C_3_N_4_ shows rod‐shaped and sheet‐like structures with a larger surface area. Figure 7B shows the photocatalytic decontamination of methyl orange dye using pristine ZnO, g‐C_3_N_4_, and ZnO‐g‐C_3_N_4_ with 10%, 20%, and 30% g‐C_3_N_4_ by weight. ZnO/g‐C_3_N_4_‐20 exhibited the highest activity. Furthermore, after five degradation cycles (Figure 7C), ZnO/g‐C_3_N_4_ maintained a decontamination rate of over 90%, showing its excellent recyclability.^[^ ^85^ ^]^

**Figure 8:**
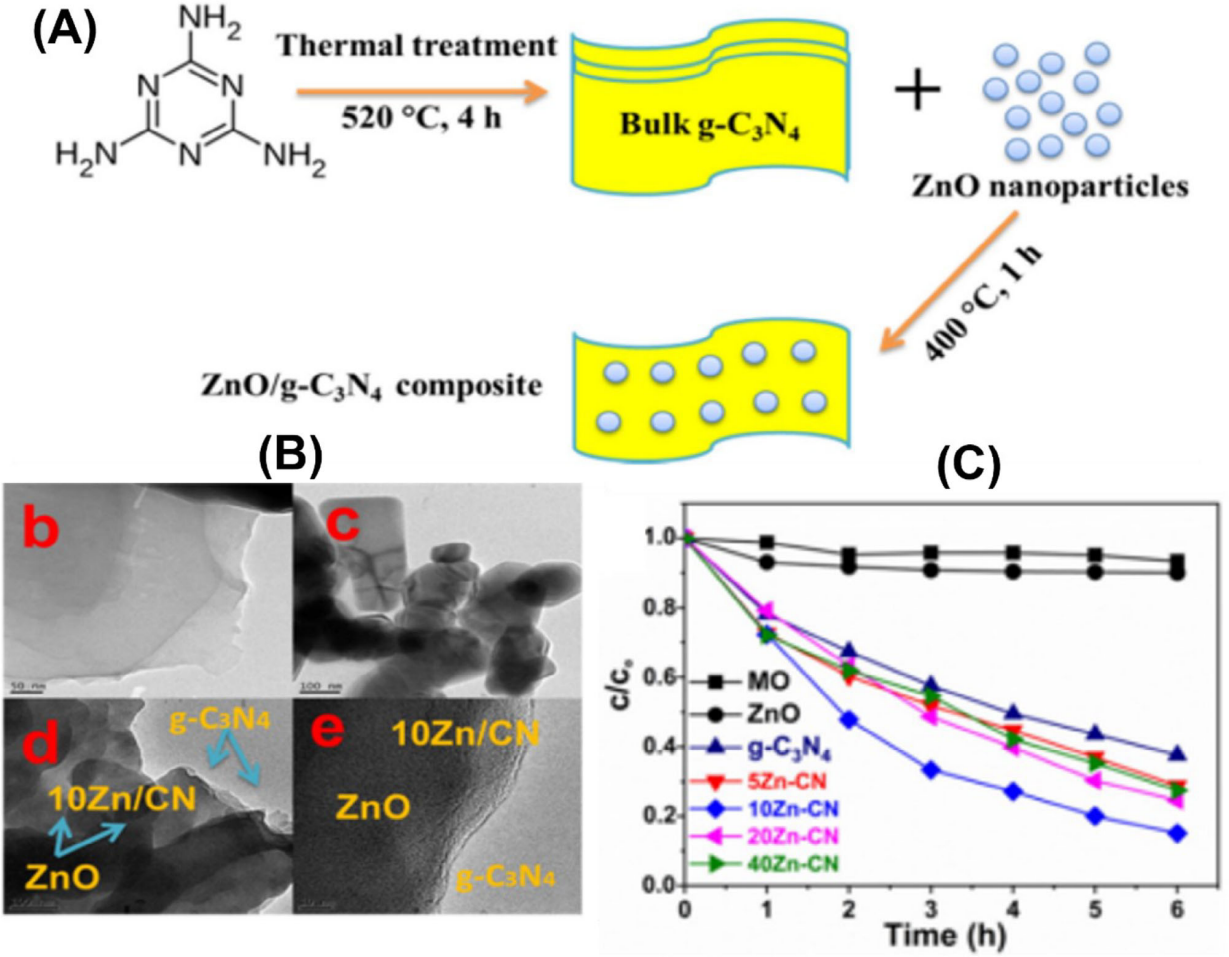
A) Synthesis of ZnO/g‐C_3_N_4_ via the calcination method. B) TEM image (b) g‐C_3_N_4_, (c) ZnO, (d) 10 Zn/CN, (e) HRTEM image of 10 Zn/CN. C) The photocatalytic degradation rate of ZnO, g‐C_3_N_4_, and (X%) Zn‐CN composites: Reproduced with permission from^[^ ^83^ ^]^Copyright 2020, Elsevier.

In addition, Ismael, M. prepared ZnO/g‐C_3_N_4_ nanocomposites by a calcination method (**Figure** **8**A). The TEM images of g‐C_3_N_4_ nanoparticles revealed a sheet‐like structure as depicted in Figure 10B (b). Figure 8B (c) corresponds to ZnO, which has a heterogeneous mixture of particles with different shapes and sizes. Correspondingly, the TEM images of the 10‐Zn‐CN composite [Figure 8B (d)] showed that the Zn particles are well distributed on the surface of g‐C_3_N_4_. This distribution is advantageous for creating a heterojunction between ZnO and g‐C_3_N_4,_ which increases charge separation, thereby increasing its photocatalytic activity. The HRTEM image of the 10‐Zn‐g‐C_3_N_4_ composite revealed the formation of a smooth interphase between ZnO and g‐C_3_N_4_ [Figure 8B (e)]. Furthermore, Figure 8C shows that the photodegradation rate of the ZnO‐g‐C_3_N_4_ composite is greater than compared of pristine ZnO and g‐C_3_N_4_. Consequently, the 10Zn‐CN composite exhibited the maximum decontamination efficiency of 88% in 6 h for MO. This outcome suggests a substantial enhancement in the photocatalytic activity of ZnO upon incorporation of g‐C_3_N_4_.^[^ ^83^ ^]^

**Figure 9:**
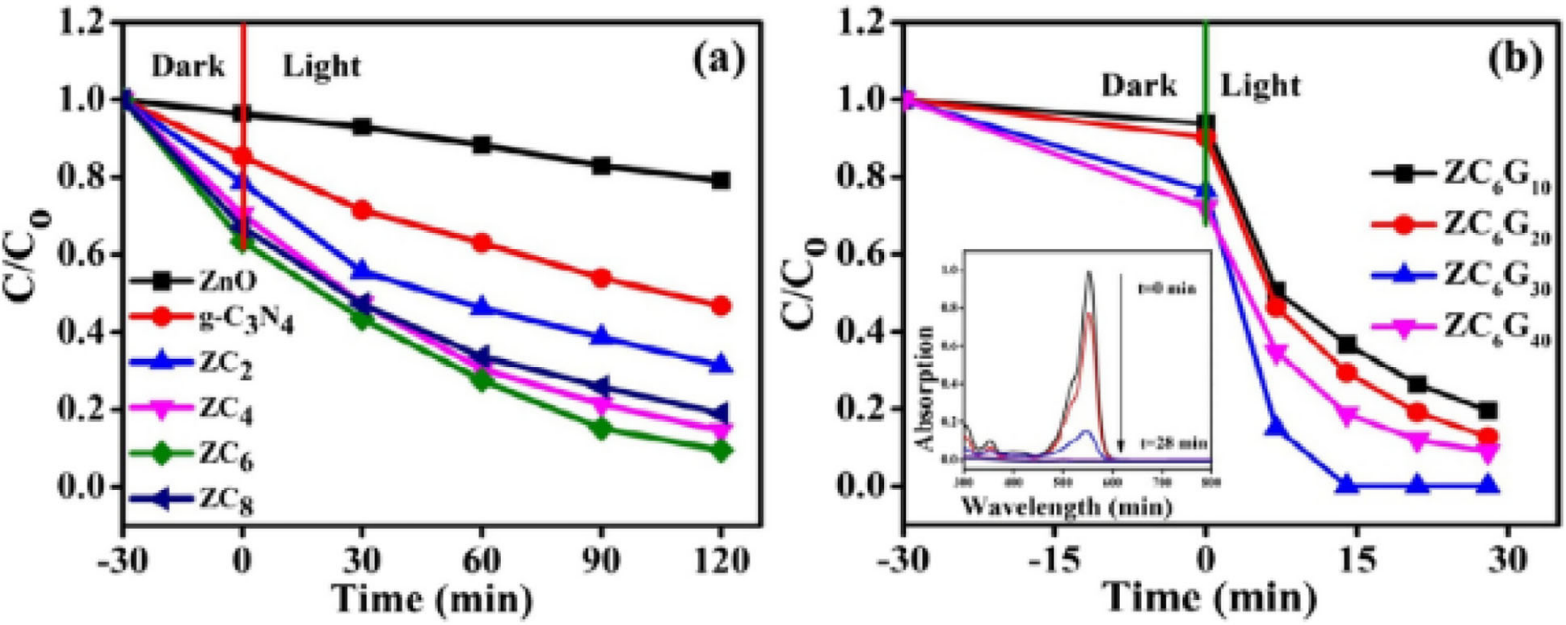
A) C/C_0_ plot for degradation of RhB for ZnO, g‐C_3_N_4,_ and ZnO‐g‐C_3_N_4_(x%). B) ZnO‐g‐C_3_N_4_(6%)‐GO(x%) in visible light irradiation. Reproduced with permission from^[^ ^89^ ^]^ Copyright 2020, Elsevier.

In addition, the development and application of carbon‐based materials for extensive use in photocatalysis has emerged as a significant topic in recent years. Graphene is a distinct form of carbon that was believed to be unstable until the first successful experimental synthesis of single‐layer graphene in 2004. It has been confirmed to be thermodynamically stable. Graphene is a 2D material with distinct features such as a higher surface area, superior electronic, mechanical, and chemical properties. Consequently, graphene is a good electron acceptor for photocatalysis because of its higher mobility and conjugated structure, which lowers the recombination rate of photoinduced electrons and holes. Thus, graphene is used in photocatalysis owing to its higher adsorption ability, greater absorption of visible light, and higher conductivity.^[^ ^86^ ^]^ Hence, the creation of graphene in heterojunctions offers an innovative dimension for enhancing photocatalytic performance. The incorporation of graphene into the ZnO‐g‐C_3_N_4_ composite presents a promising approach for enhancing photocatalytic performance. Moreover, Puri et al.,^[^ ^87^ ^]^ explained recent advancements in the utilization of graphene oxide for photocatalysis. Voon et al.,^[^ ^88^ ^]^ reviewed the synthesis of graphene‐based materials for degrading organic pollutants. Therefore, many researchers have reported that incorporating graphene oxide with ZnO‐g‐C_3_N_4_ enhances photocatalytic activity. Kumaresan et al.,^[^ ^89^ ^]^ prepared ZnO‐g‐C_3_N_4_‐GO using ultrasonication with a hydrothermal method to decontaminate the rhodamine blue dye. ZnO‐g‐C_3_N_4_ (6%) exhibited the highest RhB dye decontamination efficiency ≈90% under visible light within 120 min (**Figure** **9**A). However, ZnO‐g‐C_3_N_4_(6%)‐GO (30%) exhibited an RhB dye decontamination efficiency of ≈99% under visible light within 14 min (Figure 9B). This demonstrates a substantial reduction in the recombination of electrons and holes resulting from the incorporation of GO into ZnO‐g‐C_3_N_4_ heterojunction.

**Figure 10:**
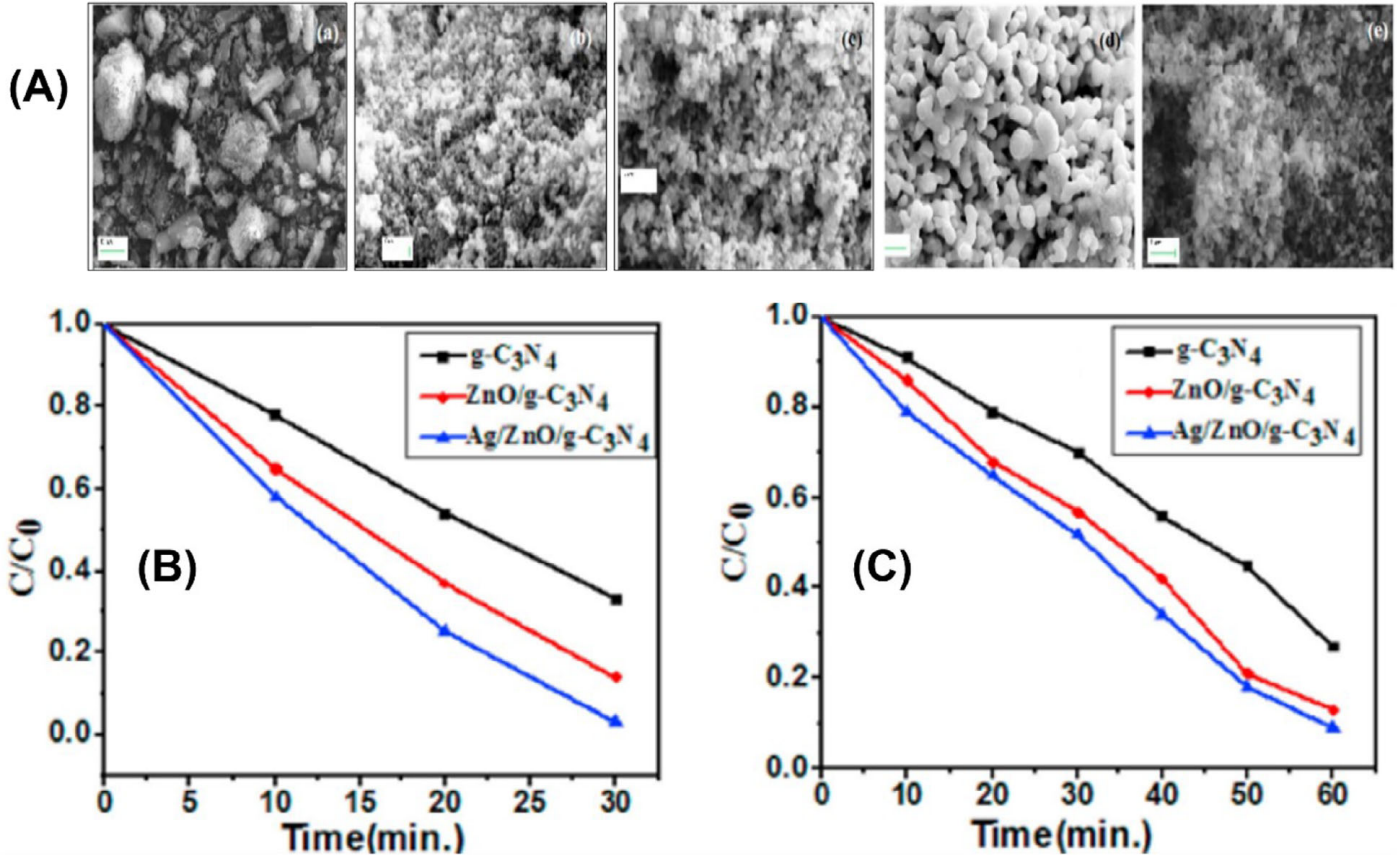
A) SEM images (a) g‐C_3_N_4_, (b)ZnO, (c)ZnO/g‐C_3_N_4_, (d) Ag/ZnO, and (e) Ag/ZnO/g‐C_3_N_4_. B) Decontamination plot of RhB and C) MG by g‐C_3_N_4_, ZnO/g‐C_3_N_4,_ and Ag/ZnO/g‐C_3_N_4_.Reproduced with permission from^[^ ^90^ ^]^ Copyright 2021, Elsevier.

Similarly, Mohanty et al.,^[^ ^90^ ^]^ incorporated Ag noble particles into ZnO/g‐C_3_N_4_ heterojunction. The co‐precipitation approach was utilized to synthesize Ag/ZnO/g‐C_3_N_4_ and investigate the photodegradation of RhB and MG under sunlight. Moreover, the SEM images [**Figure** **10**A a **]** show the irregular morphology of the pristine g‐C_3_N_4_. Figure 10A b illustrates ZnO particles with a spindle‐like morphology, and Figure 10A c shows the coverage of g‐C_3_N_4_ particles on ZnO. Notably, [Figure 10A d] shows the SEM image of Ag/ZnO, and Figure 12B (e) displays the spherical morphology of Ag/ZnO/g‐C_3_N_4_. Furthermore, (Figure 10B,C) demonstrate that Ag/ZnO/g‐C_3_N_4_ exhibited maximum decontamination efficacy for RhB and MG at 97%, and 91%respectively, in contrast to ZnO/g‐C_3_N_4_. Moreover, the efficiency of ZnO/g‐C_3_N_4_ in the photocatalytic degradation of different dyes, as reported in previous studies, is given in **Table** **1**.

**Table 1:** Percentage degradation efficiency of ZnO‐g‐C_3_N_4_ composite for the degradation of dyes.

Precursor used for the synthesis of ZnO‐g‐C_3_N_4_ composite	Method	Pollutants	Source	Degradation efficiency	Refs.
Zinc acetate and melamine	Calcination	Direct Blue	500 W Xenon and 300 W mercury lamp	99% in 100 min	[91]
Zinc acetate and melamine	One step‐Calcination	MO and p‐nitrophenol	500 W Xenon Lamp	97% in 80 min and 30% in 300 min	[92]
Urea and zinc acetate	Calcination	RhB	Visible light >400 nm	90% in 50 min	[93]
Urea and Zinc oxide	Ball milling	RhB	500 W Xenon lamp	51.3% in 120 min	[94]
Urea and Zinc oxide	Facile	MB	Visible light source	98% in 150 min	[95]
Melamine and Zinc acetate dihydrate	Hydrothermal	MB and RhB	500 W Xenon lamp	95% and 97% in 800 min	[96]
Urea and zinc carbonate	Thermal	MB	200 W Tungsten lamp	90% in 120 min	[77]
Zinc acetate, G. Mangostana pericarp extract, and melamine	Calcination	MB, Malachite green, and MO	Visible irradiation	99.16, 96.42, and 57.57 in 180 min,	[97]
Zinc acetate and urea	Calcination	MB	Visible light irradiation	99.54% in 60 min	[98]

### 5.2 Reduction of CO_2_

**Figure 11:**
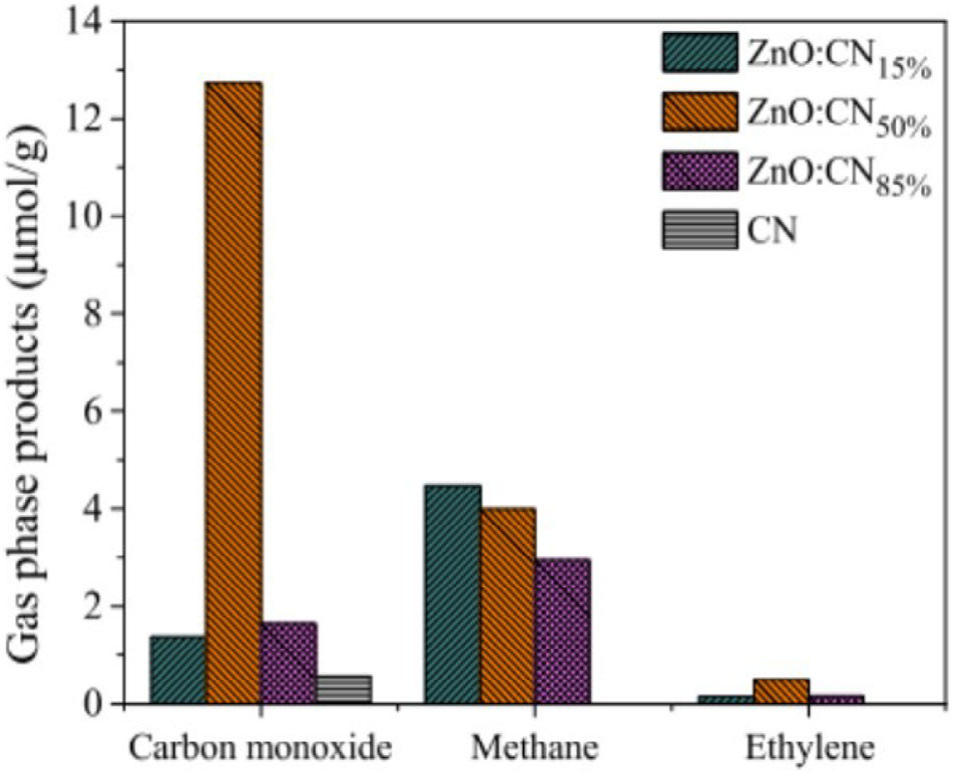
Catalytic CO_2_ photoreduction products after 6 h of reaction between CN and ZnO‐CN_Xwt%_.Reproduced with permission from^[^ ^100^ ^]^Copyright 2021, Elsevier.

Recently, chemical energy storage has become an important business sector. A critical challenge is the conversion of solar energy into chemical energy utilizing photocatalysts. This process should efficiently capture and utilize CO_2_ and solar energy and convert them into hydrocarbon fuels, thereby simultaneously addressing both energy and environmental issues.^[^ ^99^ ^]^ Several researchers have studied the reduction of CO_2_ to hydrocarbons to solve the above‐mentioned environmental problems. For instance, de Jesus Martin et al.,^[^ ^100^ ^]^ synthesized a ZnO‐g‐C_3_N_4_ nanocomposite with various percentages of g‐C_3_N_4_ (15, 50, and 85%), and observed its photocatalytic performance for reducing CO_2_. **Figure** **11** shows that the optimized ZnO‐g‐C_3_N_4_(50%) shows excellent photocatalytic performance for CO_2_ conversion into CH_4_ and C_2_H_4_, in contrast to pure ZnO and g‐C_3_N_4_. Furthermore, the synthesized heterojunction exhibited enhanced photocatalytic performance in converting CO_2_ into other products, such as CH_4_ and C_2_H_4_, owing to the fabrication of g‐C_3_N_4_ and the development of a type‐II heterojunction, which increases charge separation.^[^ ^100^ ^]^

**Figure 12:**
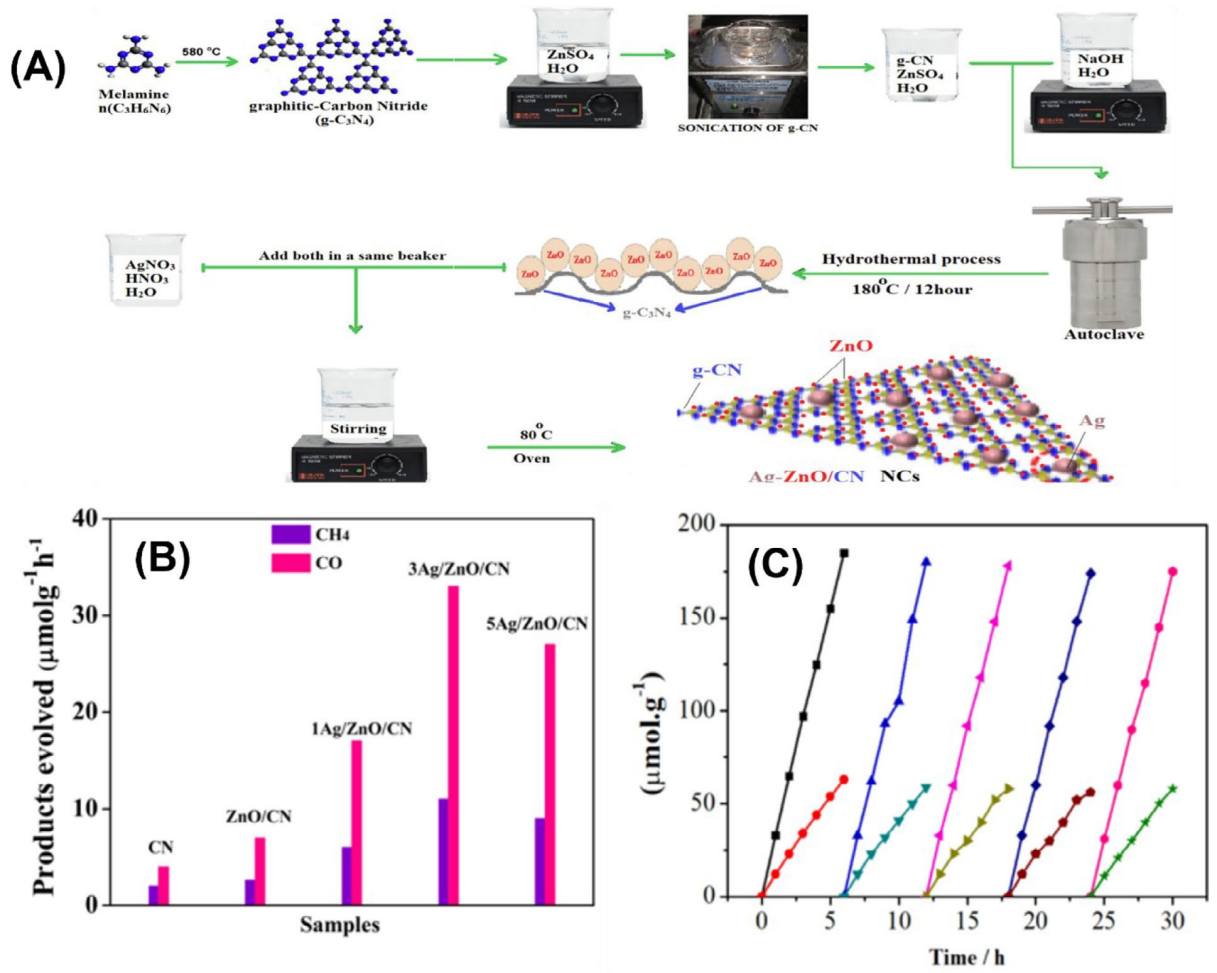
A) Schematic illustration of the synthesis of Ag‐ZnO/CN. B) Photocatalytic activity of CN, ZnO/CN, and xAg/ZnO/CN toward reduction of CO_2_. C) Recyclability and stability test by 3‐Ag/ZnO/ CN. Reproduced with permission from^[^ ^101^ ^]^ Copyright 2022, Elsevier.

Arif et al.,^[^ ^101^ ^]^ incorporated Ag particles into a ZnO‐g‐C_3_N_4_ composite, and a schematic representation of its synthesis is displayed in **Figure** **12**A. The prepared samples were studied for CO_2_ reduction into CO and CH_4_. The prepared composite exhibited significant enhancement in photocatalytic performance for the conversion of CO_2_ into CO and CH_4_ in contrast to pure g‐C_3_N_4_ (Figure 12B). Moreover, a stability test was performed, which concluded that the optimized sample 3‐Ag/ZnO/CN was stable for five successive cycles for up to 30 h (Figure 12C). Thus, a summary of the ZnO‐g‐C_3_N_4_‐based composites used for CO_2_ conversion, as reported in the previous literature, is presented in **Table** **2**.

**Table 2:** ZnO‐g‐C_3_N_4_ composites used for CO_2_ reduction.

Precursor used for the synthesis of ZnO‐g‐C_3_N_4_	Method	Source	Products [max. yield µ mol g^−1^ h^−1^]	Refs.
Zinc acetate, ethylenediamine thiourea, and melamine	Thermal	300 W Xe lamp (λ = 420 nm)	H_2_, CH_4_, and CO (22.7, 30.5, and 16.8)	[99b]
Urea and zinc nitrate hexahydrate	Calcination	300 W Xe lamp (λ = 365 nm)	CH_3_OH (0.6)	[102]
Urea, zinc acetate, hexamethylenetetramine	Calcination	300 W Xe lamp (λ = 420 nm)	CH_4_ and CO (49.7, and 13.4)	[103]
Zinc nitrate hexahydrate, Trisodium citrate, and dihydrate urea	Hydrothermal	300 W Xe lamp (λ = 420 nm)	CH_3_OH (1.32)	[104]
Zinc Acetate and Urea	Impregnation	500 W Xe lamp (λ = 420 nm)	CO (45.6)	[105]

### 5.3 Bacterial Disinfection

The wastewater contains a variety of microorganisms, including bacteria, which cause waterborne diseases like cholera, typhoid, etc. Thus, there is an urgent need to remove the bacteria from wastewater to protect the environment. Most importantly, the photocatalytic disinfection is considered an effective, sustainable, green approach compared to conventional microbial methods. Recently, the scope of g‐C_3_N_4_‐based photocatalysts has been reported for pollutant degradation and bacterial disinfection.^[^ ^106^ ^]^ Several groups have carried out the development of heterojunctions and investigated their activity for bacterial disinfection. For instance, Qamar et al.,^[^ ^107^ ^]^ reported the synthesis of g‐C_3_N_4_/ZnO/Cr using the co‐precipitation method and investigated the antibacterial activities of g‐C_3_N_4_/ZnO/Cr against Escherichia coli, Bacillus subtilis, Staphylococcus aureus, and Streptococcus salivarius. Their results confirmed that the 60%‐g‐C_3_N_4_/ZnO/Cr‐5% nanocomposite displayed a better antibacterial activity and was considered the most optimum and stable sample among its counterparts. Moreover, the increasing trend of antibacterial activity of prepared samples was arranged as g‐C_3_N_4_ < ZnO < Cr/ZnO < g‐C_3_N_4_/ZnO < g‐C_3_N_4_/ZnO/Cr. Similarly, Qamar et al.,^[^ ^108^ ^]^ reported the synthesis of g‐C_3_N_4_/ZnO/Co and investigated its antibacterial activity against gram‐positive and gram‐negative. The reported results indicate that the 60%‐g‐C_3_N_4_/ZnO/Co‐5% exhibits superior antibacterial efficiency (60%) against gram‐positive and gram‐negative bacteria as compared to its counterparts. This was due to the synergic effects of the heterojunction developed at Co‐ZnO and g‐C_3_N_4_. Adhikari et al.,^[^ ^109^ ^]^ synthesized Ag/ZnO‐g‐C_3_N_4_ using the hydrothermal method and used it for antibacterial activity against E. coli gram‐negative bacteria in the presence and absence of UV light irradiation. They showed that the antibacterial activity of Ag/ZnO‐g‐C_3_N_4_ was higher in the presence of UV light irradiation, and this was mainly due to the coupling of ZnO into g‐C_3_N_4,_ which reduces the recombination rate of electrons and hole pairs. Moreover, Sher et al.,^[^ ^110^ ^]^ prepared Mo/ZnO/g‐C_3_N_4_ nanosheets‐based nanocomposites by the co‐precipitation method and investigated them against Bacillus subtilis, Streptococcus salivarius, and Escherichia coli. Their findings revealed that Mo/ZnO/g‐C_3_N_4_ nanocomposites were the most stable sample with the highest zones of bacterial inhibition against Bacillus subtilis, Streptococcus salivarius, and Escherichia coli bacteria strains as compared to pure MoO_2_ nanoparticles. Thus, the increase in the production of reactive oxygen species within the Mo/ZnO/g‐C_3_N_4_ nanocomposites was responsible for its greater antibacterial performance.

### 5.4 Water Splitting

Recently, the usage of fuels for the production of energy due to globalization and industrialization has caused environmental pollution. Therefore, the usage of metal oxide‐based g‐C_3_N_4_ heterojunction as a photocatalyst for water‐splitting reactions involves enhancing the hydrogen evolution yield. Remarkably, the ZnO/g‐C_3_N_4_ heterojunctions are widely used as photocatalytic material for hydrogen production by water splitting reaction. For example, Grish et al.,^[^ ^74^ ^]^ demonstrated that the ZnO/g‐C_3_N_4_ heterojunctions exhibit three times higher hydrogen evolution reaction activity (1358 µmol g^−1^ h^−1^) in contrast to pristine ZnO (545 µmol g^−1^ h^−1^) and g‐C_3_N_4_ (238 µmol g^−1^ h^−1^) respectively. The increase in hydrogen evolution activity is due to the heterojunction formed between ZnO and g‐C_3_N_4_. In this direction, Vattikuti et al.,^[^ ^111^ ^]^ synthesized SnO_2_–ZnO‐g‐C_3_N_4 _and reported 1.06‐ and 2.27‐times higher hydrogen production rates (13 673.61 µmol g^−1^ h^−1^) in contrast to ZnO‐g‐C_3_N_4_ (12 785.54 µmol g^−1^ h^−1^) and pure g‐C_3_N_4_ (6017.72 µmol g^−1^ h^−1^) respectively. Similarly, Ma &Wang prepared g‐C_3_N_4_‐ZnO heterojunction and investigated its hydrogen evolution reaction activity by water splitting reaction. The results of their study concluded that g‐C_3_N_4_‐ZnO exhibited higher hydrogen evolution activity in contrast to pristine g‐C_3_N_4_. The 25%‐g‐C_3_N_4_‐ZnO displayed which is 4.6 times greater hydrogen evolution activity (306.25 µmol g^−1^ h^−1^), in contrast to pristine g‐C_3_N_4._^[^ ^112^ ^]^ Another study by Kim et al.,^[^ ^113^ ^]^ studied the photocatalytic activity of g‐C_3_N_4_/ZnO and Boron‐doped g‐C_3_N_4_/ZnO. Notably, the g‐C_3_N_4_/ZnO displayed higher hydrogen evolution activity (123 µmol g^−1^ h^−1^) as compared to pure g‐C_3_N_4_ (26 µmol g^−1^ h^−1^) and pure ZnO (8.7 µmol g^−1^ h^−1^). Moreover, the hydrogen production activity for boron doped into g‐C_3_N_4_/ZnO heterojunction was also examined. Their results concluded that boron‐doped g‐C_3_N_4_/ZnO heterojunction exhibited higher hydrogen production activity (357 µmol g^−1^h^−1^) which is 2.5‐fold times higher than g‐C_3_N_4_/ZnO.^[^ ^113^ ^]^ Similarly, Ge et al.,^[^ ^114^ ^]^ prepared g‐C_3_N_4_‐ZnO‐Au by chemical method and then utilized it for the hydrogen evolution activity. The g‐C_3_N_4‐_ZnO‐Au displayed the highest hydrogen evolution activity (46.46 µmol g^−1^ h^−1^) as compared to pristine g‐C_3_N_4_ (1.59 µmol g^−1^ h^−1^) and g‐C_3_N_4_‐ZnO (1.843 µmol g^−1^ h^−1^).

## 6 Triboelectric Feature for Catalysis and Environmental Remediation

Tribocatalysis is an emerging field that combines mechanical processes, electrochemistry, and catalytic chemistry, presenting an innovative approach to catalysis.^[^ ^115^ ^]^ Tribocatalysis is also referred to as mechano‐electrocatalysis by some researchers, as this involves frictional force interaction between the reactant and the catalyst, initiating the conversion of mechanical energy into electrical energy. This conversion is important for initiating the catalytic reactions, making tribocatalysis an effective and eco‐friendly approach for environmental remediation.^[^ ^116^ ^]^ The removal of wastewater pollutants through the tribocatalysis effect has gained significant interest due to its superior advantages of recyclability and upscalability. Several tribocatalysts, such as BiVO_4_,^[^ ^116a^ ^]^ ZnO,^[^ ^117^ ^]^ CdS,^[^ ^118^ ^]^ TiO_2_,^[^ ^119^ ^]^ and Bi_2_WO_6_^[^ ^120^ ^]^ have already been reported for degrading the organic pollutants.

**Figure 13:**
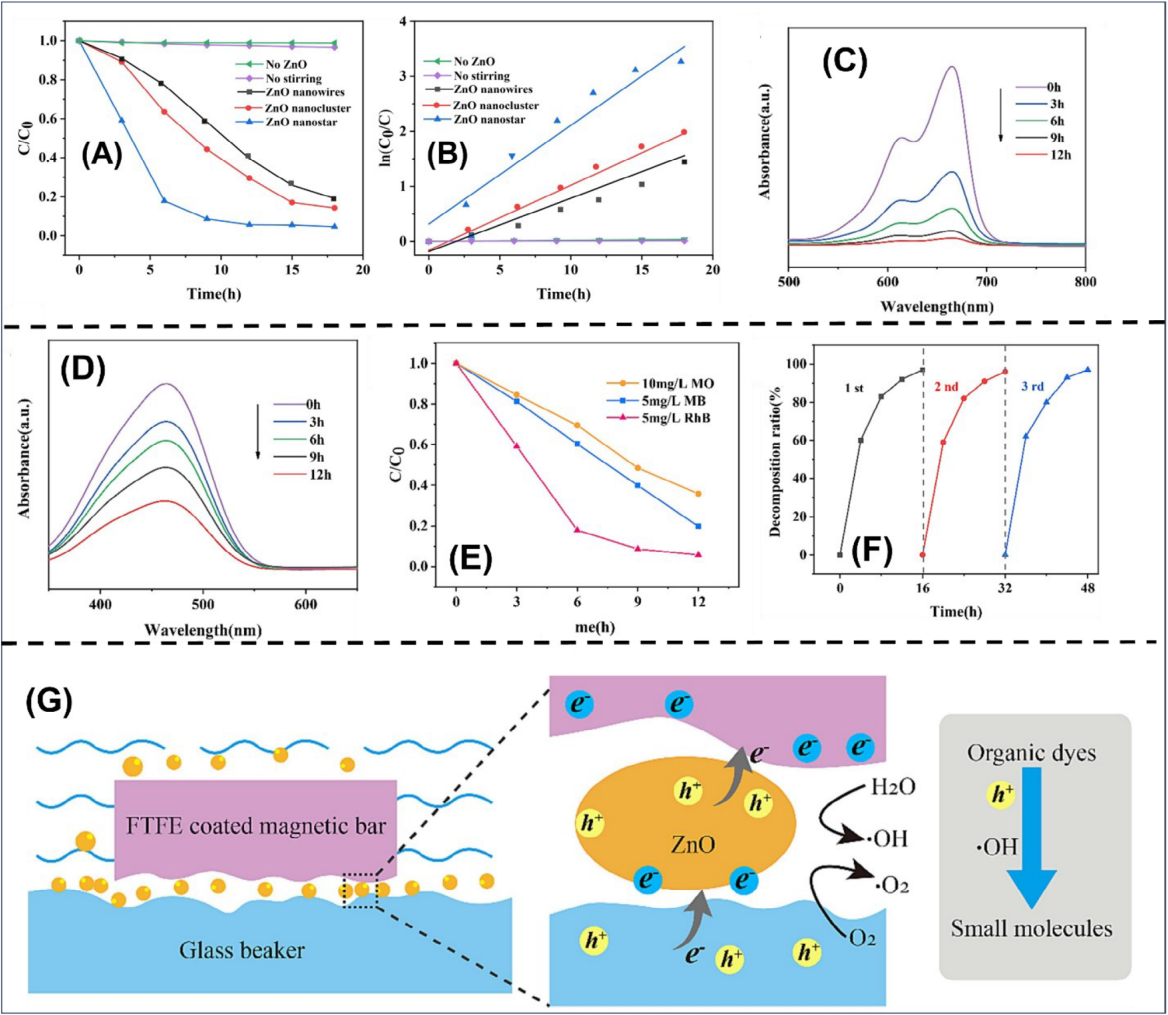
A) Degradation of RhB dye by ZnO with different morphologies under magnetic stirring, B) kinetic analysis, C) degradation of dyes by magnetic agitation in darkness, D) adsorption spectrum of MB dye solution after magnetic stirring of ZnO nanostars, E) degradation of MO, MB, and RhB dyes concentration with time. F) Degradationrate of RhB for three successive cycles, G) mechanism of tribocatalysis degradation of RhB by ZnO tribocatalyst. Reproduced with permission from^[^ ^121^ ^]^ Copyright 2024, Elsevier.

Recently, Chong et al.,^[^ ^121^ ^]^ have reported the synthesized nanowires, nano‐stars, and nanoclusters from ZnO to study the shape‐dependent tribocatalysis effect with respect to the degradation of organic dyes (**Figure** **13**). The friction‐induced catalytic performance of ZnO nanostructures was investigated under magnetic agitation, which promoted the decontamination of rhodamine B (RhB) dye in the dark. In the absence of a catalyst under magnetic stirring, no significant degradation of RhB dye was observed. The ZnO nanostars (under magnetic agitation) displayed the highest degradation efficiency (80%) in 9 h, which further exceeded 95% after 12 h of friction. This further demonstrated the importance of magnetic stirring and ZnO nanomaterials in tribocatalysis. The higher surface area provides greater active sites for pollutants to adsorb, which leads to greater separation of photogenerated electrons and hole pairs, causing redox reaction and improving tribocatalytic efficiency (Figure 13A). It was revealed that the kinetic rate constants of ZnO nanoclusters (1.88 min^−1^) and nanowires (1.4 min^−1^) were significantly higher than ZnO nanostars (0.003287 min^−1^) (Figure 13B). Moreover, the degradation of methyl orange (MO) and methylene blue (MB) dyes was also investigated to study the effect of ZnO nanoclusters in tribocatalysis. Figure 13C,D shows that the absorption peaks of MB dye (664 nm) and MO dye (464 nm) decreased with time. Notably, Figure 13E revealed the degradation rate of MO (72%), MB (93%), and RhB (95%) of ZnO nanoclusters under tribocatalytic conditions. These variations in degradation efficiency arise due to the different redox chemical bonds. It was observed that the degradation efficiency remained consistent through the three consecutive cycles, confirming that ZnO nanoclusters exhibit the highest reusability and stability for the decontamination of RhB dye (Figure 13F). Furthermore, the mechanism of tribocatalysis degradation of RhB dye by ZnO nano stars has also been illustrated in Figure 13G).^[^ ^121^ ^]^ **Table** **3** illustrates the % degradation efficiency of some reported tribocatalysts used in environmental remediation.

**Table 3:** Percentage degradation efficiency of some reported tribocatalysts used in environmental remediation.

Tribocatalyst	Pollutant	Tribocatalytic degradation efficiency	Refs.
BiVO_4_	MB	69% in 1440 min.	[116a]
CDS	RhB	98% in 420 min.	[118]
TiO_2_	MV and RhB	94.7 and 99.7% in 200 min.	[119]
SrTiO_3_	RhB	88.6% in1440 min.	[122]
ZnO	RhB	99% in 90 min.	[117]
Bi_12_TiO_20_	RhB	98%in 720 min.	[123]

## 7 Photodegradation Kinetics

Pollutant degradation occurs through the breakdown of hydroxyl radicals (OH**^.^**). These radicals are produced through the interaction of holes and electrons excited to the valence and conduction bands of the photocatalyst under irradiation. Therefore, the reaction rate (Equation 1) can be represented as: (P: pollutants, I: irradiation, and k_r_: constant).

(1) $$Rate\propto Irradiation{\left(O_{2}\right)}_{adsorbed}{\left(P\right)}_{adsorbed}$$

Under constant irradiation and partial pressure of oxygen, the rate of reaction is expressed as (Eqs. 2 and 3):

(2) $$Rate=k_{r}{\left(P\right)}_{adsorbed}$$

Or

(3) $$Rate=k_{r}{\left(P\right)}_{aq}$$

The following two equations illustrate the Langmuir‐Hinshelwood and Eley‐Rideal mechanisms. The concentration of adsorbed dyes can be represented through different adsorption isotherms like Langmuir, Freundlich, and Temkin. Conversely, the rate equation for pollutant concentration in an aqueous solution can be expressed by the following equations for 1st and 2nd order reactions (Eqs. 4 and 5), respectively.^[^ ^124^ ^]^

(4) $$ln\frac{{\left[P\right]}_{o}}{{\left[P\right]}_{t}}=k_{1}t$$

(5) $$\frac{1}{P_{t}}=k_{2}t+\frac{1}{P_{0}}$$

According to Nygullie et al.,^[^ ^125^ ^]^ degradation of MB follows a pseudo‐first‐order reaction as per the given Equation (6):

(6) $$ln\frac{{\left[C\right]}_{o}}{{\left[C\right]}_{t}}=kt$$

where C_o_ and C_t_ are the initial and final concentrations of the methylene blue dye, respectively, and k is the rate constant. The results of their study showed that the (75%) ZnO/g‐C_3_N_4_ has a higher rate constant value (0.0128min^−1^) and a sixfold increase in photocatalytic activity compared to pristine ZnO, g‐C_3_N_4_ and (60%) ZnO/g‐C_3_N_4_, and (70%) ZnO/g‐C_3_N_4,_ respectively.^[^ ^125^ ^]^ Similarly, P. Meena et al.,^[^ ^98^ ^]^ demonstrated in their research work that the photodegradation of MB by ZnO/g‐C_3_N_4_ follows pseudo‐first‐order reactions. They concluded that ZnO/g‐C_3_N_4_ (25%) exhibits a higher rate constant for the decontamination of MB dye that is 7.7‐fold greater than that of pristine ZnO nanorods and g‐C_3_N_4_ nanosheets. A higher surface area, a greater number of active sites, and reacting species played an important role in increasing the photocatalytic performance of the composites.^[^ ^98^ ^]^ Furthermore, Liu et al.,^[^ ^126^ ^]^ demonstrated that ZnO‐g‐C_3_N_4_(10%) exhibited a higher rate constant value (0.0 02965 min‐^1^), which is two times greater than that of pure ZnO. It was also concluded that the degradation of RhB follows a first‐order reaction kinetics.

## 8 Comparative Analysis of ZnO‐Based Materials with Other Oxides

The ZnO‐based materials have attained significant attention in environmental remediation. However, other semiconductor oxides‐based photocatalysts such as TiO_2_,^[^ ^127^ ^]^ MoS_2_,^[^ ^128^ ^]^ WO_3_,^[^ ^129^ ^]^ and Bi_2_O_3_^[^ ^130^ ^]^ also demonstrate substantial potential for the treatment of environmental pollutants. The coupling of semiconductor oxides with other semiconductors to form a heterojunction is an effective approach to enhance the photocatalytic activity. Remarkably, several researchers reported the importance of semiconductor‐based photocatalysts for environmental remediation. For instance, Zhang et al.,^[^ ^128^ ^]^ reported the synthesis of TiO_2_/MoS_2_ and investigated it for the degradation of methyl orange dye under visible light irradiation. Their findings indicate that the TiO_2_/MoS_2_ exhibited higher photocatalytic activity (90%) as compared to pure TiO_2_ and MoS_2_. Similarly, Liu et al.,^[^ ^129^ ^]^ demonstrated the synthesis of TiO_2_/WO_3_ and utilized it for the photodegradation of methylene blue dye. It was concluded that 1% mol monolayer‐WO_3_/TiO_2_ displayed 98.05% photocatalytic degradation efficiency for methylene blue light in 10 min as compared to pure TiO_2_ under solar light irradiation.^[^ ^129^ ^]^ Moreover, a study by Alothman et al.,^[^ ^127^ ^]^ demonstrated that TiO_2_/g‐C_3_N_4_ exhibited 97.20% and 89.62% photocatalytic degradation efficiency for RO‐16 and RhB dyes in 90 min. However, TiO_2_‐based photocatalysts also showed higher photocatalytic activity for the degradation of organic pollutants, but ZnO‐based photocatalytic materials showed remarkable potential in environmental remediation. For instance, ZnO/g‐C_3_N_4_ exhibited 99.54% photocatalytic degradation efficiency for methylene blue in 60 min under visible light irradiation.^[^ ^98^ ^]^ Similarly, in another study by Wang et al.,^[^ ^93^ ^]^ reported that ZnO/g‐C_3_N_4_ showed 90% photocatalytic degradation efficiency for Rhodamine B in 50 min. Furthermore, **Table** **4** illustrates the photocatalytic degradation efficiency of widely studied semiconductor photocatalysts in environmental remediation.

**Table 4:** Photocatalytic degradation efficiency of reported semiconductor photocatalysts in environmental remediation.

Photocatalyst	Pollutant	Source	Photocatalytic degradation efficiency	Refs.
TiO_2_/g‐C_3_N_4_	RO‐16 and RhB	Solar Light	97.20% and 89.62% in 90 min.	[127]
TiO_2_/MoS_2_	MO	Xenon Lamp	90%% in 10 min.	[128]
TiO_2_/WO_3_	MB	Solar Light	98.05% in10 min.	[129]
Bi_2_O_3_/g‐C_3_N_4_	RhB	Xenon Lamp	98% in 80 min.	[130]
ZnO/TiO_2_	MB	Visible Light	98%in 50 min.	[131]

### 8.1 Conclusion and Future Perspectives

Wastewater is now a major issue in the 21st century, causing different types of hazardous diseases. Heavy metal ions and organic contaminants in wastewater discharged from different industries usually harm the ecological environment. Some are naturally degraded, but others require appropriate treatment because they cannot be naturally degraded. It is necessary to handle textile wastewater to avoid queries associated with its pollution. Therefore, semiconductor‐based heterogeneous photocatalysis is highly effective in eliminating contaminants present in wastewater, without producing toxic by‐products. Notably, the ZnO‐g‐C_3_N_4_ displays excellent photocatalytic performance, making it a highly auspicious candidate for environmental applications. This review focuses on different methods for increasing the photocatalytic activity of pure ZnO, particularly focusing on coupling with g‐C_3_N_4_ to produce ZnO‐g‐C_3_N_4_ heterojunctions. Consequently, ZnO‐based nanocomposite photocatalysts have shown significant results in the treatment of environmental pollutants compared to pristine ZnO. Previous studies have confirmed that the introduction of g‐C_3_N_4_ increases the efficacy of pure ZnO. Most importantly, the ZnO‐g‐C_3_N_4_ composite is an effective and eco‐friendly material for the removal of environmental contaminants. Moreover, the incorporation of carbon‐based materials such as graphene oxide and noble metals like Ag has significantly enhanced the photocatalytic activity of ZnO‐g‐C_3_N_4_ heterojunction. Furthermore, this review summarizes various studies on the treatment of environmental contaminants. Finally, the mechanism of ZnO‐g‐C_3_N_4_ in the decontamination of contaminants is explained. However, some future approaches have been suggested to enhance the photocatalytic activity of ZnO‐g‐C_3_N_4_‐based composites.

One of the most important applications of the ZnO‐g‐C_3_N_4_‐based composites is photocatalysis. The composite exhibits promising features like higher visible light absorption, charge separation, and large surface area, which make it effective for degrading contaminants from the environment. Therefore, future research is required to optimize the composition and surface characteristics of the composites to further increase their activity. Although ZnO‐g‐C_3_N_4_‐based composites have been extensively investigated for different photocatalytic applications, several challenges still remain. One of the challenges is to develop a simpler synthetic approach that does not require a harsh environment, thereby enhancing the stability and structure of these materials. Therefore, there is an urgent need to develop a simpler and more eco‐friendly approach to enhance the surface area of these materials. Finally, many scientists use dyes as representative contaminants for photocatalytic decontamination. However, dyes are easier to decontaminate than other pollutants, such as pesticides. Thus, there is an urgent need to deepen our understanding of the degradation mechanism and the interaction of contaminants with the prepared photocatalyst.

## Conflict of Interest

The authors declare no conflicts of interest.

## Author Contributions

H.A. performed conceptualization, data curation, formal analysis, validation, visualization, writing‐original draft, writing – review and editing. M.S. performed visualization, writing – review and editing. F.S.M. performed (conceptualization, visualization, writing review, and editing). M.K.I. performed visualization, writing – review and editing. T.L. performed funding acquisition, visualization, conceptualization, writing – review and editing, and supervision. Y.K.M. performed conceptualization, funding acquisition, investigation, project administration, resources, supervision, visualization, writing‐original draft, writing‐ review and editing.
